# Biological substantiation of antipsychotic-associated pneumonia: Systematic literature review and computational analyses

**DOI:** 10.1371/journal.pone.0187034

**Published:** 2017-10-27

**Authors:** Janet Sultana, Marco Calabró, Ricard Garcia-Serna, Carmen Ferrajolo, Concetta Crisafulli, Jordi Mestres, Gianluca Trifirò’

**Affiliations:** 1 Department of Biomedical and Dental Sciences and Morpho-functional Imaging, University of Messina, Messina, Italy; 2 Department of Medical Informatics, Erasmus Medical Centre, Rotterdam, Netherlands; 3 Chemotargets SL, Parc Científic de Barcelona (PCB), Baldiri Reixac Barcelona, Catalonia, Spain; 4 Department of Experimental Medicine, Unit of Clinical Pharmacology, Campania Regional Centre of Pharmacovigilance and Pharmacoepidemiology, University of Campania “L. Vanvitelli”, Naples, Italy; 5 Research Group on Systems Pharmacology, Research Program on Biomedical Informatics (GRIB), IMIM Hospital del Mar Medical Research Institute and University Pompeu Fabra, Parc de Recerca Biomèdica de Barcelona (PRBB), Catalonia, Spain; Chiba Daigaku, JAPAN

## Abstract

**Introduction:**

Antipsychotic (AP) safety has been widely investigated. However, mechanisms underlying AP-associated pneumonia are not well-defined.

**Aim:**

The aim of this study was to investigate the known mechanisms of AP-associated pneumonia through a systematic literature review, confirm these mechanisms using an independent data source on drug targets and attempt to identify novel AP drug targets potentially linked to pneumonia.

**Methods:**

A search was conducted in Medline and Web of Science to identify studies exploring the association between pneumonia and antipsychotic use, from which information on hypothesized mechanism of action was extracted. All studies had to be in English and had to concern AP use as an intervention in persons of any age and for any indication, provided that the outcome was pneumonia. Information on the study design, population, exposure, outcome, risk estimate and mechanism of action was tabulated. Public repositories of pharmacology and drug safety data were used to identify the receptor binding profile and AP safety events. Cytoscape was then used to map biological pathways that could link AP targets and off-targets to pneumonia.

**Results:**

The literature search yielded 200 articles; 41 were included in the review. Thirty studies reported a hypothesized mechanism of action, most commonly activation/inhibition of cholinergic, histaminergic and dopaminergic receptors. In vitro pharmacology data confirmed receptor affinities identified in the literature review. Two targets, thromboxane A2 receptor (TBXA2R) and platelet activating factor receptor (PTAFR) were found to be novel AP target receptors potentially associated with pneumonia. Biological pathways constructed using Cytoscape identified plausible biological links potentially leading to pneumonia downstream of TBXA2R and PTAFR.

**Conclusion:**

Innovative approaches for biological substantiation of drug-adverse event associations may strengthen evidence on drug safety profiles and help to tailor pharmacological therapies to patient risk factors.

## Introduction

Antipsychotics drugs (APs) have been associated with several adverse reactions including stroke, acute kidney disease and pneumonia, especially in aged patients [1–3]. In 2009 the Food and Drug Administration issued a warning on the increased risk of death when APs were used off-label in persons with dementia, pneumonia being among the most commonly reported causes of death. Many observational studies have evaluated the association between APs and the risk of community or hospital-acquired pneumonia in the elderly population [4–14]. However, most studies investigated APs by class rather than by individual drug, with a few exceptions. In this respect, classifying drugs as conventional or atypical is a traditional perspective still reminiscent of the one drug–one target paradigm [15]. It is now widely recognized that most drugs bind with different affinities to multiple proteins, including but not limited to traditional receptor targets [16], a property often referred to as polypharmacology [17].This makes each single drug essentially unique pharmacologically.

The biological pathways underlying antipsychotic-induced pneumonia are not currently known, although several plausible hypotheses have been postulated [18]. Understanding which drugs are associated with the highest risk of pneumonia would allow prescribers to select drugs with a lower risk of pneumonia in persons with risk factors predisposing them to infectious respiratory diseases. Such risk factors include being bedridden, having chronic respiratory diseases and/or being prescribed sedating drugs. There is emerging data from observational studies suggesting a different safety profile for individual APs, which may be missed when studying drug effects by class [19, 20]. The potential effects of such differences in adverse drug reactions (ADRs) among single APs could in turn have an impact on AP persistence and healthcare utilization as a result of ADRs, or relapse of symptoms because of differential AP drug discontinuation. While observational studies are a crucial starting point for the identification of increased risk of adverse drug reactions they cannot point to an underlying biological mechanism. Nevertheless, identifying a biologically plausible cause for drug-related risk, i.e. signal substantiation, is essential to confirm the validity of such risk and identify new off-targets that otherwise cannot be predicted on the basis of ‘traditional’ systems pharmacology, i.e. cholinergic, histaminergic, or serotonergic systems etc. Signal substantiation is defined as any activity aiming to provide a biological explanation of why an ADR occurs[21]. The rationale behind this is that an ADR must be biologically plausible to increase the feasibility of a causal relationship between drug and ADR, in particular for type A ADRs, i.e. those which are dose-dependent and are related to the drug’s known pharmacological activity.

The aims of this study were to: 1) conduct a systematic review aiming to identify all hypothesized mechanisms of AP-associated pneumonia in published literature, including studies employing any study designs (including case reports), in which persons of any age used APs for any indication, provided that the outcome was pneumonia; 2) confirm the most relevant drug targets potentially involved in AP-associated pneumonia using computational approaches for drug safety profiling [22], as well as to identify novel drug targets, with the ultimate goal of extrapolating clinically relevant information.

## Methods and materials

### Literature review

Medline and Web of Science were searched for literature from their inception to 27^th^ February 2017 using the following terms: “pneumonia” and “antipsychotic agents”. Articles were excluded if they were not published in English language or if the full text of the article was not accessible even after contacting the author. Eligibility criteria included treatment with APs for any indication in persons of any age and pneumonia as the outcome under investigation. All study designs and article types were eligible for inclusion, including case reports, letters to the editor, observational studies and systematic reviews/meta-analyses. For observational studies reporting a risk of pneumonia with AP use, the study design, population studied, exposure and comparator, outcome investigated, hypothesized mechanism underlying AP-associated pneumonia and risk estimate was reported. Two investigators independently examined the titles and abstracts of selected articles and obtained full texts of potentially relevant papers. Disagreement on inclusion was resolved by discussion. The findings were reporting accordingly the PRIMA checklist (S1 Fig).

### Screening of known and predicted antipsychotic binding to receptors and associated safety events

CT-link software [22] was used to analyze the experimentally known pharmacology and its potential link to known safety outcomes of AP drugs. Known in vitro pharmacology data was extracted from ChEMBL [23]. Statistically significant protein signatures associated to each safety outcome were generated by analyzing the confusion matrix resulting from the number of drugs with affinity above or below a certain affinity threshold for a given target and associated, or otherwise, to a given safety event. Protein signatures were defined for 1,455 safety terms, mapped to Unified Medical Language System (UMLS) codes, and classified in 25 toxicity categories [24]. CT-link was screened for targets and safety outcomes associated with a set of 7 APs commonly investigated in the relation to the risk of pneumonia, namely, amisulpride, clozapine, haloperidol, olanzapine, quetiapine, risperidone, and zotepine [25–29].

### Pathway construction

Once targets of interest were identified in CT-Link (i.e., novel targets), the pathways that could potentially explain a novel biological link between AP off-targets, i.e., binding targets other than intended ones, and safety terms related to pneumonia were identified and investigated using the following software: Cytoscape [30], the GeneMania [31], ClueGO [32] and CyTarget Linker [33] plug-ins (S3 Fig). Cytoscape software was used to find group of elements, either genes or proteins, that interact with the user-defined query (which consists of one or more genes/proteins), representing them graphically. Each of the elements (either genes or proteins) found by Cytoscape are referred as “nodes” (graphically represented by circles). The interactions are referred as edges (graphically represented by lines connecting different nodes).

To construct a possible molecular pathway downstream of antipsychotic binding to its off-targets, two approaches were used. In the first approach, a “Physical Interactions” database was used [31] to identify possible proteins and/or metabolic products that may be responsive to the function of the identified proteins to which AP drugs bind. Cytoscape was used to identify all known nodes which physically interact with AP off-targets, using data inputs from the “Physical Interactions” database of geneMania plug-in. This step was iterated, using the results of the previous step as input, to widen the biological net of physical interactors.

In the second approach, the “Pathway Commons” [31] database was used in order to identify nodes related to the function of the off-targets identified. For this step, Cytoscape was further screened to identify the pathways that are functionally linked with AP protein targets and pneumonia-related symptoms. The “Pathway Commons” database of the geneMania plug-in was used in this step. All molecular cascades, including those weakly related to our starting query, were identified. Nodes not related to the phenotype under investigation were removed manually.

### Selection and clustering of candidate genes from the biological net identified: Mapping of hypothetical molecular cascades leading to AP-derived pneumonia due to novel targets

The relevance of the interactors to pneumonia-related symptoms was checked through the use of Medline and Cytoscape built-in databases. The nodes having a stronger association were highlighted. These are hereafter referred to as associated nodes(AN). Nodes within the biological net, linking the associated nodes to identified AP off-targets were clustered, where a cluster is defined as a subgroup of nodes within the biological net under investigation that share a particular characteristic. In this case, the common characteristic is that they are related to both AP targets and the associated nodes found in the biological net. ClueGo [32] and CluePedia [34] plug-ins were then used to identify pathways involved in the function of the nodes included in the clusters.

## Results

### Literature review

**Fig 1:**
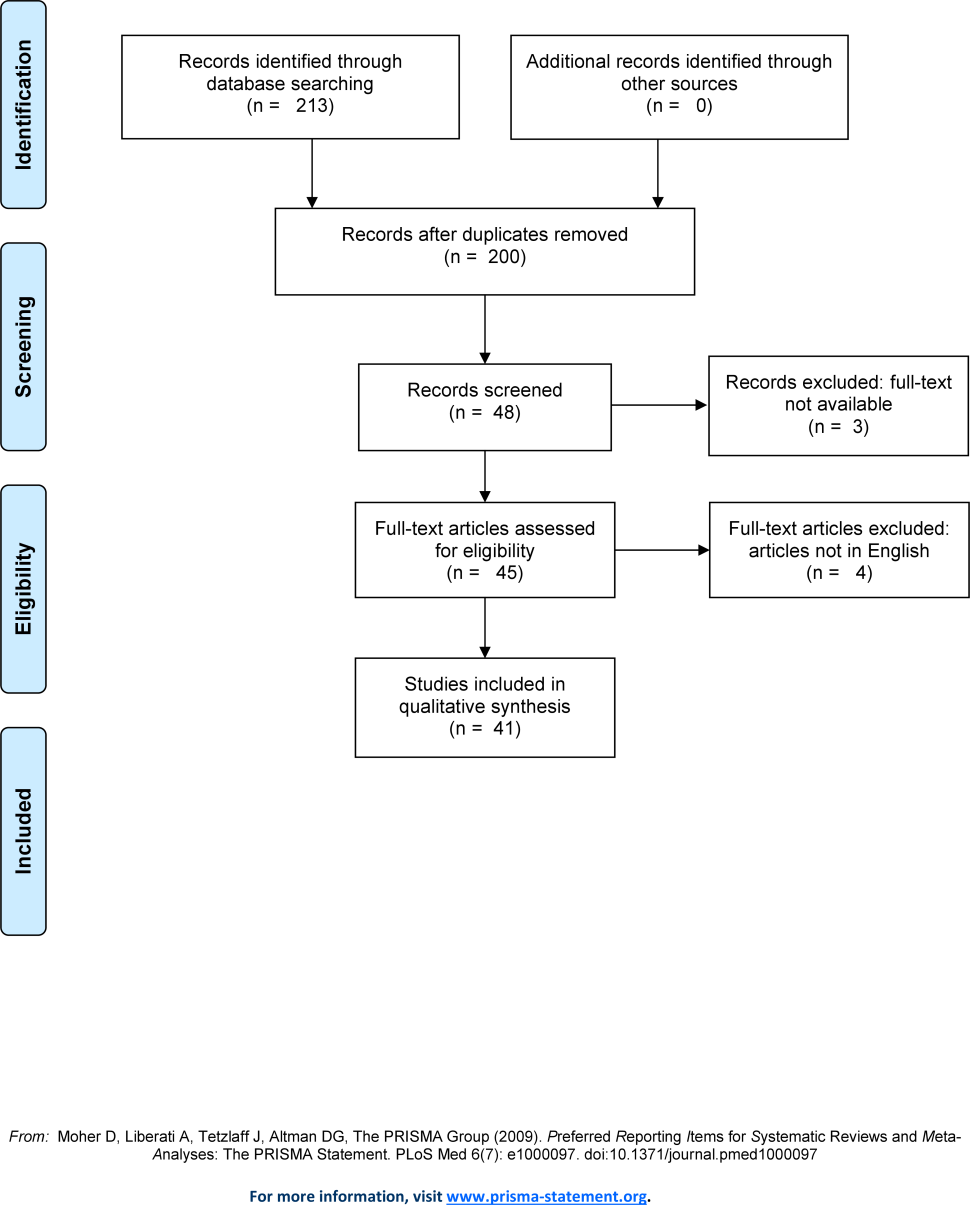
Selection of publications in Medline and Web of Science, using the PRISMA model.

The results of the article selection process are reported in Fig 1. The search in Medline and Web of Science yielded 200 articles. Of these, only 48 articles were deemed suitable for further consideration as they specifically concerned APs as drugs of interest which may have led to pneumonia. Seven articles were subsequently excluded because they were not in English or had no full text available, even after the authors were contacted (see S1 Table for details on excluded studies with no full text). Forty-one articles were included in the final assessment and of these, the majority were observational studies (N = 16) [1, 4, 6–10, 12, 13, 18, 25–29, 35–46]. Of the 41 articles included, 30 hypothesized a mechanism of action. Ten studies were reviews/meta-analyses [1, 13, 18, 27, 41–46], while other studies included 12 case reports [47–58]and one letter to the editor[15]. Most of the literature concerned patients aged 65 and older [1, 4, 6, 8–10, 12, 18, 35, 38, 41, 45, 47, 49, 51] while others investigated an adult population with bipolar/schizophrenic disorders [26, 36, 37, 40, 48, 50, 55, 56, 59]. No randomized clinical trials (RCTs) were identified, but one meta-analysis of 6 RTCs was found [27](S2 Fig). Limited information on the effect of dose and duration regarding the risk of pneumonia was available, although some studies suggested an increased risk of pneumonia with higher AP doses [4, 29] and mostly close to the start of treatment [29], while another study found no clear link between the duration of AP exposure and risk of pneumonia[39]. Some groups of AP-treated persons were found to be at higher risk of pneumonia, including persons with Parkinson’s disease [25], schizophrenia [25], and those concomitantly using multiple antipsychotic drugs [26].

The most widespread theories about the biological mechanisms underlying pneumonia due to antipsychotic use concern the affinity of the antipsychotics to cholinergic [1, 4, 6, 8, 9, 12, 18, 25, 26, 36, 37, 43, 45, 48, 51, 59], histaminergic [4, 6, 9, 25, 26, 43–45, 59] and dopaminergic [1, 6, 9, 18, 25, 36, 41–44, 49, 58]receptors (Table 1). The clinical events triggered by APs binding to their targets and leading to pneumonia are summarized in Table 1.These include dry mouth and impaired peristalsis due to muscarinic M1 receptor blockade, as well as sedation with subsequent impaired swallowing due to histamine H1 receptor blockade. Lesser known mechanisms proposed involved the immune system [6, 10, 26, 36, 41, 43], cytochrome metabolism system [47], inflammatory pathways [15, 41], an eotaxin (i.e., a chemoattractant for eosinophils) and serotonin eosinophilic-specific chemo-attracting action leading to eosinophilic pneumonia [49].

**Table 1:** Summary of potential biological mechanisms underlying antipsychotic-associated pneumonia identified in the literature.

Receptor system	Molecular/cellular target	Associated signs/symptoms	Antipsychotic drug or drug class
Cholinergic system	M2, M3 and M5 receptor antagonism [59]	-	Clozapine
	M4 muscarinic receptor agonism[48, 59]	Hypersalivation[37, 59]	Clozapine
	M1 muscarinic receptor antagonism [26, 59]	Xerostomia[26, 36, 37]	Clozapine
		Xerostomia[36]	Olanzapine
		Esophageal dilatation and hypomotility[26, 36, 37]	Clozapine
		Esophageal dilatation and hypomotility[36]	Olanzapine
		Impaired peristalsis[36]	Olanzapine
		Impaired peristalsis [36, 37]	Clozapine
	Interaction with muscarinic receptor system with no specification as to receptor subtype and nature of effect (agonist or antagonist) [1, 9, 12, 18, 43]	Not specified	Not specified
	Anticholinergic activity with no specification to receptor subtype [8, 25]	Xerostomia[4, 6, 43, 45]	Atypical APs
	Anticholinergic activity with no specification to receptor subtype [45]	Aspiration[4, 8, 45]	Conventional APs
	Anticholinergic activity with no specification to receptor subtype[4]	Esophageal dysfunction [8]	Atypical APs
		Decreased peristaltic function[8]	Not specified
		Bronchorrhea[51]	
		Hypersalivation[51]	
		Hypersalivation [37]	Clozapine
Adrenergic system	Alpha-2 receptor antagonism [48]	Not specified	Clozapine
Serotonergic system	5-HT_2a_ receptors antagonism [49]	Not specified	Risperidone
Dopaminergic system	Post-synaptic D_2_ receptors antagonism [49]	Not specified	Risperidone
	D_2_ receptors[36]	Extrapyramidal symptoms[36]	Haloperidol
	Dopaminergic activity with no specification to receptor subtype and nature of effect (agonist or antagonist)[9, 25, 43]	Extrapyramidal symptoms[4, 9, 25, 45]	Conventional APs
	Dopaminergic activity with no specification to receptor subtype and nature of effect (agonist or antagonist)[1, 41–44]	Extrapyramidal symptoms [30–33]	Not specified
		Dysphagia[29, 43]	Not specified
		Oropharyngeal dyskinesia[6, 43]	
		Oropharyngeal rigidity and spasm[6, 43]	
		Aspiration[6, 58]	
		Gasping and choking[58]	
Histaminergic system	H_1_ receptor blockade[45]	Sedation [4, 6, 9, 25, 26, 43–46]	Conventional APs
	H_1_ receptor blockade [6, 25, 43, 44, 46]	Impaired swallowing [4, 6, 9, 25, 43, 45]	Atypical APs
	H_1_ receptor blockade[26]	Aspiration[4, 37, 43, 45]	Clozapine, olanzapine, and quetiapine
	H_1_ receptor blockade [43, 44]	Not specified	Phenothiazines
		Not specified	Not specified
	H_1_ receptor blockade[59]	Impaired esophageal peristalsis, due to sedation [59]	Clozapine
	Effect on H_1_ receptors, with no specification as to nature of effect (agonist or antagonist)	Sedation[29]	Not specified
	No subtypes specified	Sedation[29, 36, 37]	Olanzapine, clozapine
-	Immune system[29]	Agranulocytosis[10]	Not specified
		Altered cytokine profile [41]	Individual APs or class not specified
		Agranulocytosis[6, 26, 43, 59]	Clozapine
-	No pharmacologic or other systems specified	Sedation[1, 10, 41–43]	Not specified
		Sedation[48]	Clozapine
		Sedation[8]	Atypical APs
		Impaired swallowing and dysphagia [10, 41–43, 54]	Not specified
		Impaired swallowing and dysphagia[28]	Atypical APs
		Impaired swallowing and dysphagia[15, 48]	Clozapine
		Xerostomia[10, 42]	Not specified
		Hypersalivation[15, 48, 50]	Clozapine
		Esophageal dysfunction [15, 50]	Clozapine
		Suppression of cough reflex [41, 54]	Not specified
		Sialorrhea [53]	Clozapine
		Hypertonic movement of pharyngeal muscles [54]	Not specified

Abbreviations- AP: antipsychotic.

Most of the hypotheses concerned antipsychotic drug classes rather than individual drugs, or referred to antipsychotics in general. Several studies did not specify a biological mechanism leading to pneumonia but described the antipsychotic-associated clinical signs or symptoms which may cause pneumonia [10, 15, 35, 42, 44, 50, 53, 54]. The most commonly hypothesized physiological/clinical conditions leading to antipsychotic-associated pneumonia were extrapyramidal symptoms (5 studies reporting extrapyramidal symptoms with conventional APs, 5 studies where AP class was not specified; none for atypical APs), sedation (7 studies reporting sedation with conventional APs, 5 with atypical APs, 6 where class was not specified), xerostomia (6 studies reporting xerostomia after atypical AP use, 2 studies not specifying AP drug class and none for conventional APs) and oropharyngeal dyskinesia/impaired swallowing (10 studies reporting this for atypical APs, 8 without specifying a drug class; none for conventional APs). Less common clinical features included aspiration, sialorrhea, agranulocytosis, and suppression of the cough reflex.

Of all the observational studies/reviews identified, 17 reported a risk estimate of pneumonia associated with antipsychotic use (Table 2). Most risk estimates were available for AP drug classes rather than individual drugs, suggesting that the overall risk of pneumonia associated with atypical APs is similar to conventional APs, when non-use of APs was the reference group. No comparison of pneumonia risk could be made for individual APs due to the variety of reference groups used.

**Table 2:** Summary of all identified observational studies investigating the risk of antipsychotic-associated pneumonia.

Type of study	Study population	Exposure	Mechanisms hypothesized	Outcome and risk estimate (95% CI)	High risk groups
Nested case-control study[25]	Persons aged ≥65	Olanzapine, risperidone, quetiapine, ziprasidone, aripiprazole	The increased risk of pneumonia with olanzapine compared to quetiapine could be due to the increased affinity of olanzapine to histaminergic and muscarinic receptors compared to quetiapine. Pneumonia may be mediated via extrapyramidal effects, sedation, impaired swallowing.	Pneumonia risk (quetiapine as comparator) Risperidone HR_Adj_ = 1.14 (1.10–1.18) Olanzapine HR_Adj_ = 1.10 (1.04–1.16) Ziprasidone HR_Adj_ = 0.97 (0.81–1.16) Aripiprazole HR_Adj_ = 0.92 (0.84–1.00)	Parkinson’s disease
Systematic review and meta-synthesis of observational studies [41]	Persons aged ≥65	Conventional or atypical APs	The risk of pneumonia among conventional APs may be mediated by extrapyramidal effects, leading to pharyngeal rigidity and dysphagia, and sedation, in turn leading to suppression of the cough reflex and a higher risk of aspiration. APs may also modulate cytokine levels and immune response but it is not clear how this may differ between conventional and atypical agents.	Average pneumonia relative risk and range (atypical APs as comparator) RR = 1.01 (0.84–1.28)	None specified
Retrospective cohort study[9]	Long-term care residents aged ≥65	Conventional or atypical APs	Conventional APs may cause dysphagia. Their affinity to dopaminergic receptors causes extrapyramidal adverse effects that can lead to pneumonia. Both atypical and conventional APs may have significant anticholinergic effects which increase the risk of pneumonia. Histamine H_1_ receptor blockade may lead to sedation, which may be associated with dysphagia and possibly, aspiration pneumonia.	Risk of pneumonia (atypical APs as comparator) HR = 1.24(0.94–1.64)	None specified
Nested case-control study[6]	Persons aged ≥65	Any AP or APs by class	Pneumonia may be due to aspiration, dysphagia and/or impaired cough reflex. Dopaminergic blockade may lead to dyskinesia of the oro-pharyngeal muscles, rigidity and spasm of the pharyngeal muscles which increases the risk of aspiration. The blocking of dopamine receptors may also result in hyperfunctional involuntary movements (dyskinesia) of the oral pharyngeal musculature, rigidity, and spasm of the pharyngeal musculature, which can result in aspiration. Another mechanism involves dryness of the mouth that leads to impaired oropharyngeal bolus transport. Furthermore, sedation is also a well-known cause of swallowing problems, in particular caused by histamine-1-receptor blocking in the central nervous system. Some APs are known to have direct or indirect effects on the immune system. In less than 1% of treated patients clozapine may cause agranulocytosis, which increases the risk of infections.	Risk of pneumonia with any AP use(non-use as comparator) Current use (0 to 7 days) OR_Adj_ = 1.6 (1.3–2.1) Recent past use (8–30 days) OR_Adj_ = 0.89 (0.5–1.6) Past use (>31 days) OR_Adj_ = 0.69 (0.5–1.0) Risk of pneumonia by AP class (non-use as comparator) Use of atypical APs OR_Adj_ = 3.1 (1.9–5.1) Use of conventional APs OR_Adj_ = 1.5 (1.2–1.9) Use of both classes OR_Adj_ = 1.9 (0.5–7.4)	None specified
Nested case-control study[26]	Persons with a schizophrenia diagnosis	Conventional APs by class; atypical APs by class and individually	Pneumonia may occur due to AP drugs binding to histaminergic-1 (H1) receptors and muscarinic-1 (M1) receptors. Atypical APs with a strong binding affinity for histaminergic-1 (H1) receptors or with significant cholinergic activity may increase the risk of pneumonia.	Risk of hospitalization for pneumonia by drug class and for individual class (non-use as comparator) RR_Adj_ for any current use of atypical APs = 1.69 (1.43–2.01) RR_Adj_ for current clozapine use = 3.18 (2.62–3.86) RR_Adj_for current olanzapine use = 1.83 (1.48–2.28) RR_Adj_ for current quetiapine use = 1.63 (1.31–2.04) RR_Adj_ for current zotepine use = 1.48 (1.15–1.91) RR_Adj_ for current risperidone use = 1.32 (1.12–1.56) RR_Adj_ for current amisulpride use = 1.14 (0.79–1.65) RR_Adj_ for any current use of conventional APs = 1.38 (0.92–2.07)	Persons prescribed clozapine concomitantly with olanzapine, quetiapine, risperidone, quetiapine, zotepine or amisulpride; persons prescribed clozapine, risperidone, quetiapine, olanzapine or zotepine concomitantly with other non-clozapine APs
Systematic review and meta-analysis[44]	Persons of all ages	Any AP drug; meta-analysis conducted for conventional antipsychotics or atypical antipsychotics compared to non-use	Extrapyramidal effect may mediate aspiration pneumonia.	Risk of pneumonia (non use as comparator) OR for conventional AP use = 1.68 (1.39–2.04) OR for atypical AP use = 1.98 (1.67–2.35)	Chronic obstructive pulmonary disease, asthma, diabetes mellitus, congestive heart failure, smoking, malnutrition
Case control study[8]	Hospitalized persons aged ≥65	Atypical APs (clozapine, olanzapine, quetiapine, aripiprazole, ziprasidone, and risperidone)	AP -associated esophageal dysfunction can cause pneumonia. Sedation due to anticholinergic effects decreases peristaltic function increasing the risk of aspiration.	Risk of community acquired pneumonia (use of atypical APs in persons who did not develop pneumonia as comparator) OR_Adj_ = 2.26 (1.23–4.15)	Chronic obstructive pulmonary disease, asthma, diabetes mellitus, congestive heart failure, smoking
Nested case-control[4]	Persons ≥65 years	Atypical or conventional AP use	Conventional APs may lead to extrapyramidal effects as akinesia which increase the risk of aspiration. Pneumonia may also be due to anticholinergic receptor blockade that causes xerostomia and impaired peristalsis and H1-receptor blockade that causes sedation.	Risk of fatal or non-fatal pneumonia (past use as comparator) OR_Adj_for current atypical antipsychotic use = 2.61 (1.48–4.61) OR_Adj_ for current conventional antipsychotic use = 1.76 (1.22–2.53) OR_Adj_ for current butyrophenone use = 1.57 (1.06–2.30) OR_Adj_ for current phenothiazine use = 4.16 (1.46–11.87) OR_Adj_forcurrent use of other conventional antipsychotics = 2.26 (1.21–4.24) OR_Adj_for current risperidone use = 3.51 (1.94–6.36) OR_Adj_for current olanzapine use = 1.90 (0.61–5.90) OR_Adj_for current of piamperidone = 1.55 (1.00–2.43) OR_Adj_for current use of haloperidol = 1.95 (1.20–3.17) OR_Adj_for current use of zuclopenthixol = 2.25 (1.00–5.08)	Use of ≥0.15 defined daily dose for both drug classes
Retrospective cohort study[38]	Persons with dementia aged ≥65	Conventional or atypical APs	None specified	Risk of pneumonia for conventional APs (atypical APs as comparator) OR_Adj_ = 1.57 (1.05–2.34)	None specified
Retrospective cohort study[12]	Nursing home residents aged ≥65years	Psychotropic drugs: antidepressants, atypical APs, benzodiazepines, conventional APs.	Anticholinergic effects may give rise to pneumonia	Risk of pneumonia with conventional APs (atypical APs as comparator) RR_Adj_ = 0.94 (0.56–1.58)	None specified
Meta-analysis of 6 double-blind phase II and III trials investigating the use of risperidone in Alzheimer’s disease patients compared to placebo[27]	Alzheimer’s disease patients	Risperidone	None specified	Risk of pneumonia as cause of death with risperidone compared to placebo % deaths = 1.0 (0.38–1.60)	None specified
Retrospective cohort study[7]	Elderly persons admitted to hospital for pneumonia	Conventional or atypical APs	None specified	Risk of in-hospital death for pneumonia patients (i.e. fatal pneumonia) (non-use as comparator) Conventional AP OR_Adj_ = 1.51 (1.04–2.19) Atypical AP OR_Adj_ = 1.20 (0.96–1.50)	None specified
Retrospective cohort study[36]	Persons with a diagnosis of bipolar disease	Conventional or atypical APs or single individual APs	Olanzapine and clozapine may cause pneumonia through an anticholinergic effect at M1 receptors, inducing dry mouth, esophageal dysfunction and impaired peristalsis. Both these drugs may also cause sedation through an antihistaminergic effect. Haloperidol may increase the risk of pneumonia through extrapyramidal symptoms mediated by dopamine-2 receptors.	Risk of pneumonia (non-current use as comparator) RR_Adj_ for any atypical AP = 2.07(1.58–2.71) RR_Adj_ for clozapine = 2.59 (1.90–4.66) RR_Adj_ for olanzapine = 2.97 (1.90–4.66) RR_Adj_ for quetiapine = 2.12 (1.48–3.03) RR_Adj_ for zotepine = 1.52 (0.98–2.38) RR_Adj_ for risperidone = 1.74 (1.21–2.50) RR_Adj_ for conventional AP = 2.32 (1.76–3.05) RR_Adj_ for chlorpromazine = 1.10 (0.68–1.78) RR_Adj_ for haloperidol = 3.68 (2.66–5.09) RR_Adj_ for sulpiride = 1.29 (0.94–1.76)	None specified
Retrospective cohort study[37]	Persons with a diagnosis of schizophrenia	Conventional or atypical APs or single individual APs	Clozapine may cause pneumonia through an M1 receptors blockade, inducing dry mouth, esophageal and impaired peristalsis; clozapine may paradoxically also cause excessive salivation due to disrupted cholinergic function which increases the risk of pneumonia	Risk of pneumonia (non-current use of APs, i.e. use > 30 days of the date of pneumonia) RR_Adj_ for conventional APs = 0.97 (0.75–1.25) RR_Adj_ for chlorpromazine = 0.83 (0.48–1.44) RR_Adj_ for haloperidol = 1.11 (0.80–1.52) RR_Adj_ for flupenthixol = 0.88 (0.43–1.44) RR_Adj_ for sulpiride = 0.96 (0.70–1.33) RR_Adj_ for atypical APs = 0.92 (0.72–1.18) RR_Adj_ for clozapine = 1.40 (1.05–1.88) RR_Adj_ for olanzapine = 1.09 (0.71–1.67) RR_Adj_ for quetiapine = 1.08 (0.73–1.59) RR_Adj_ for zotepine = 0.79 (0.46–1.37) RR_Adj_ for risperidone = 0.62 (0.46–0.84) RR_Adj_ for amisulpride = 1.11 (0.61–2.01) RR_Adj_ for aripiprazole = 0.71 (0.33–1.54)	Cancer, cardiovascular disease, asthma, anti-inflammatory medications
Self-controlled case series[39]	Elderly persons with pneumonia	Conventional or atypical APs	None specified	Risk of pneumonia within the first week after AP initiation(non-exposure as comparator) RR_Adj_ for conventional APs = 2.07 (1.45–2.95) RR_Adj_ for atypical APs = 1.92 (1.44–2.56)	None specified
Retrospective cohort study [28]	Elderly persons who underwent a coronary artery by-pass graft	Conventional or atypical APs; olanzapine, quetiapine or risperidone vs. haloperidol in sensitivity analysis	Atypical APs can cause aspiration pneumonia by impairing swallowing.	Risk of pneumonia after cardiac surgery (conventional AP as comparator) RR for atypical APs after propensity score matching = 1.11 (0.89–1.38) Risk of pneumonia after cardiac surgery (haloperidol as comparator) RR for quetiapine after propensity score matching = 0.99 (0.76–1.29) RR for risperidone after propensity score matching = 1.19 (0.91–1.56) RR for olanzapine after propensity score matching = 1.11 (0.83–1.47)	None specified
Retrospective cohort study [29]	Persons with Alzheimer’s disease and persons with no Alzheimer’s disease	Any AP use; risperidone, quetiapine and haloperidol	AP drugs can cause pneumonia as a result of extrapyramidaleffects, swallowing impairment, and sedation, caused by action on the dopaminergic, cholinergic, and histaminergic systems. Otherpossiblemechanismsmayoccurthrough the immune system.	In persons with AD, risk of pneumonia (non-use as comparator) HR_Adj_ for any AP use = 3.43 (2.99–3.93) HR_Adj_ for quetiapine (risperidone as comparator) = 0.77 (0.58–1.03) HR_Adj_ for haloperidol (risperidone as comparator) = 1.90 (1.25–2.90)	Increasing dose, initial phases of treatment

Abbreviations- AP: antipsychotic; Adj: adjusted; CI: confidence intervals; HR: hazard ratio; OR: odds ratio; RR: risk ratio.

### Off-target pharmacology and links to respiratory safety events

**Fig 2:**
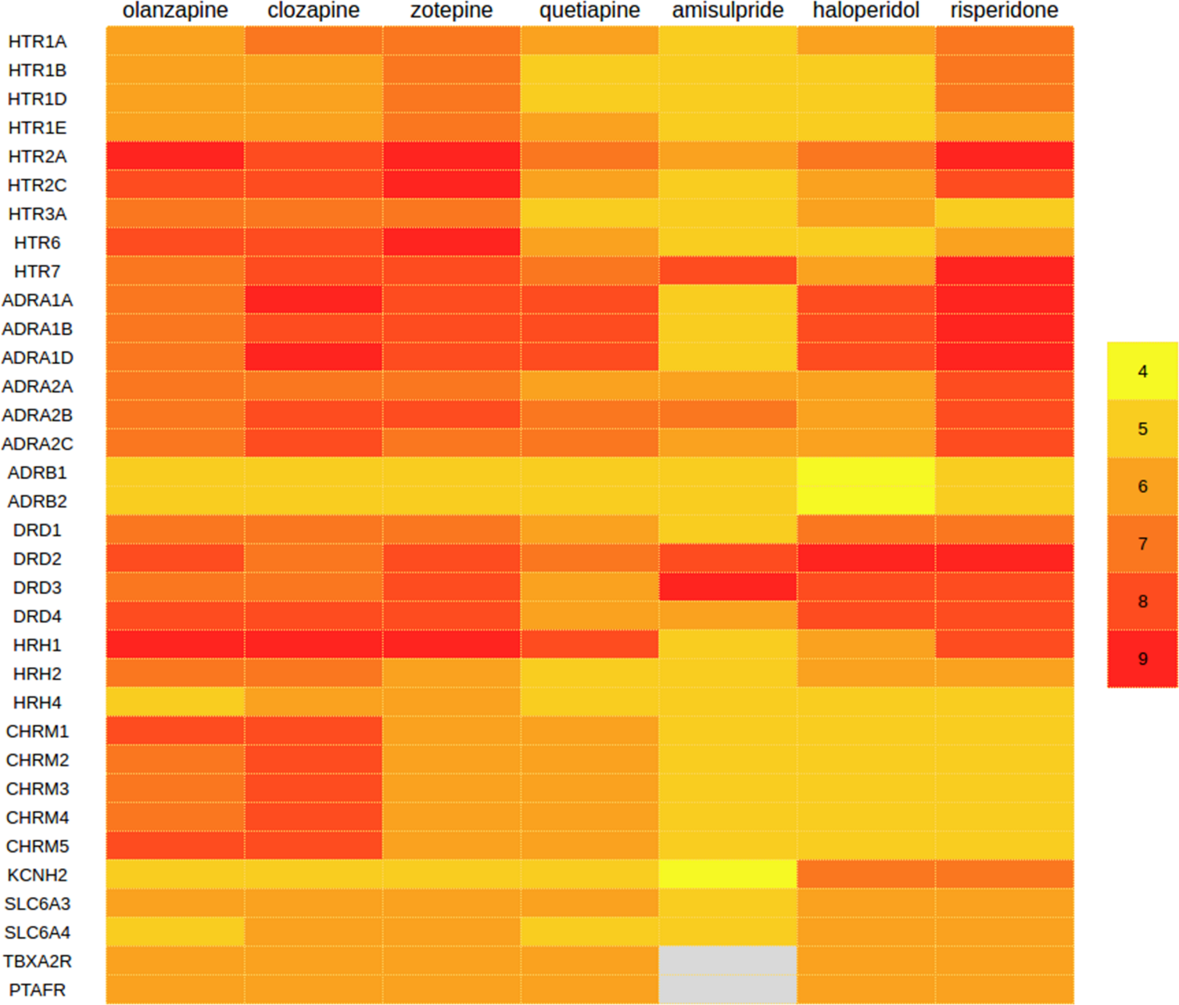
The polypharmacology of 7 commonly studied antipsychotics in the context of pneumonia, across 34 proteins for which experimentally known pK_i_ values are available. The affinities for the receptors TRXA2R and PTAFR concern predicted, not experimentally known, values; these are presented here for comparison purposes. Abbreviations: HTR- serotonin receptors; ADR: adrenergic receptors; DRDs- dopamine receptors; HRHs: histamine receptors; CHRs- muscarinic receptors; KCNH2: hERG transporter, SLC6A3: dopamine transporter, SLC6A4: serotonin transporter; TBXA2R- thromboxane A2 receptor; PTAFR- platelet activating factor receptor. Color coding reflects the experimentally known pK_i_ values, yellow being inactive (pK_i_ = 4; K_i_ = 100 nM), red being highly active (pK_i_ = 9; K_i_ = 1 nM), and grey meaning that no data is available for that interaction.

The results of the search in CT-link for the known AP receptor-binding profile broadly confirmed the results of the literature review (Fig 2). The comparison of the experimental affinity profiles of the 7 APs across a set of proteins for which in vitro affinity data was available shows that, even though these drugs are all considered to belong to the same pharmacological class, there are significant differences between their pharmacologic profiles. For example, amisulpride and haloperidol show a clear binding preference for the dopamine receptors D2 (DRD2) and D3 (DRD3), while having a much weaker affinity for the histamine H1 receptor (HRH1) than the other APs. In addition, with the exception of quetiapine and amisulpride, all APs bind strongly to the serotonin receptor subtypes 5-HT_2A_ (HTR2A) and 5-HT_2C_ (HTR2C). Also, in general, clozapine and risperidone seem to interact more strongly with the alpha-2A adrenergic receptors than the other APs. Finally, olanzapine and clozapine interact markedly with the muscarinic receptors compared to other APs. Altogether, the unique polypharmacology of every individual antipsychotic will ultimately translate in a unique drug safety profile associated with those protein interactions.

Most drugs are the result of a long and careful drug design process aiming at maximizing the affinity of a drug for its primary intended target, in the case of antipsychotics, dopaminergic receptor blockade. The primary targets of a drug are therefore those proteins for which the drug has strongest affinity. Accordingly, a total of 41 protein interactions with binding affinity stronger than 10nM (pKi ≥ 8.0) are currently experimentally known for those 7 APs [23]. Of these, 39 interactions (95%) are with aminergic G protein-coupled receptors. They will be most likely the main factor responsible for some of the antipsychotic-associated pneumonias reported. However, in spite of the optimization process, most drugs will still have residual, weaker, affinities for other proteins. In the case of the 7 APs in this study, the number of known protein interactions goes up to 184 when considering weaker binding affinities, such as 1μM (pKi ≥ 6.0). This off-target pharmacology, i.e. drugs targets other than the known and/or expected ones, could also contribute and be partly responsible for some of the respiratory safety events linked to pneumonia. The identified off-targets as potentially novel mediators of AP-associated pneumonia were then screened using a repository of drugs and their associated safety events. The thromboxane A2 receptor (TBXA2R) and the platelet-activating factor receptor (PTAFR) were preliminarily identified as such novel off-targets of interest for which micromolar affinity was observed for all 7 APs except for amisulpride.

A repository of the data from drug labels, safety predictions and ontologies in CT-link was used to screen safety events associated with the set of 7 APs. It was found that a total of 95 respiratory safety terms were associated with these antipsychotics. The most commonly identified terms were alveolitis, lung diseases, dyspnea, and respiration disorders (S2 Table). The antipsychotics most consistently linked to pneumonia-related signals were quetiapine and risperidone, whereas the least associated with pneumonia-related signals were olanzapine and clozapine. Interestingly, the latter were the two antipsychotics having a receptor binding profile markedly different from the other APs in their preferred binding for muscarinic receptors (Fig 2).

Among the off-targets identified, further analysis was carried out for the two potentially novel proteins involved in AP-associated pneumonia, TBXA2R and PTAFR. Drug safety events (including respiratory events) in CT-link are linked to data on the receptors potentially associated with that safety event. Pneumonia in relation to the antipsychotics screened was indeed found to be associated with TBXA2R and PTAFR.

### Biological pathway construction

**Fig 3:**
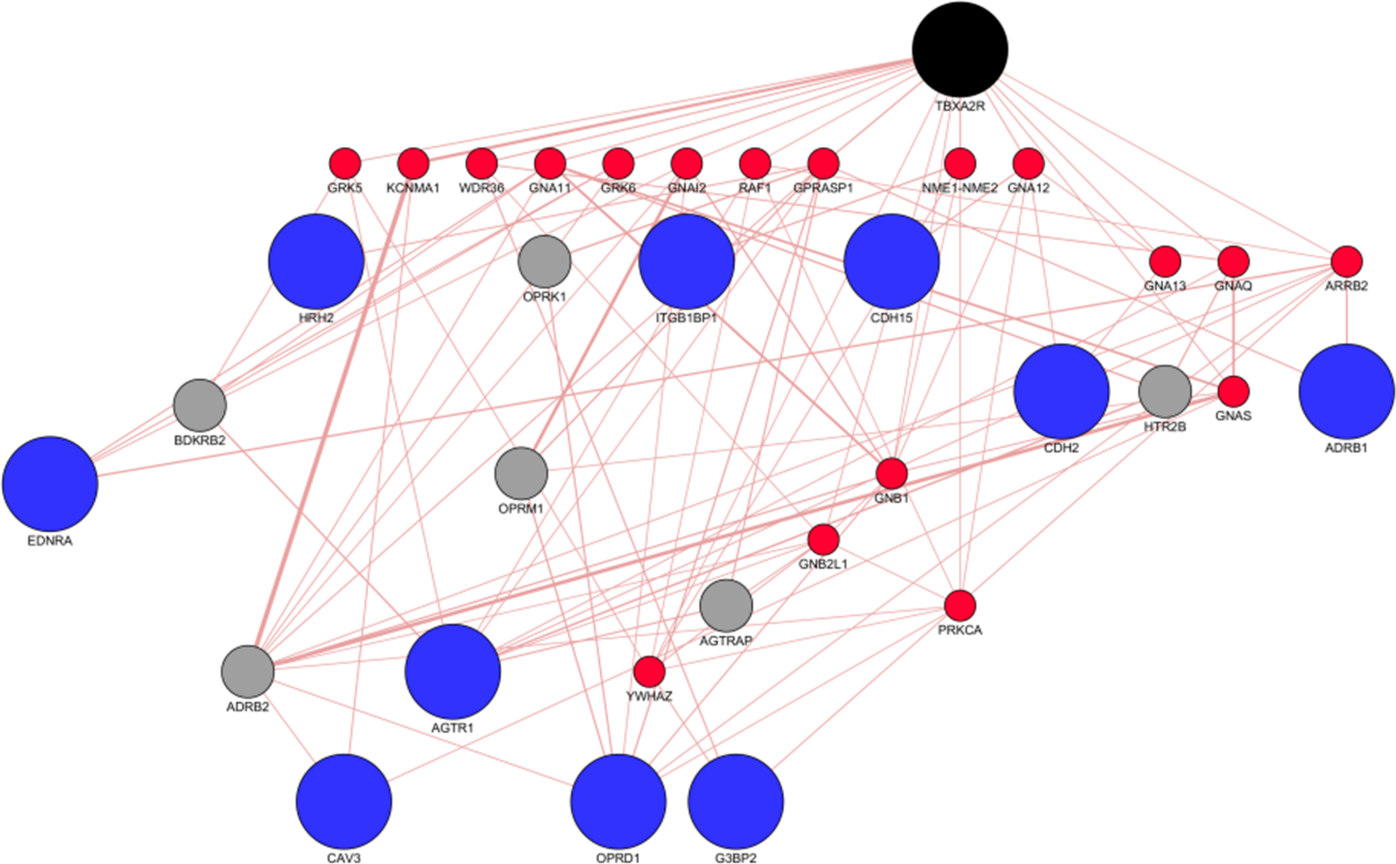
Clusters identified for investigation for TBXA2R. TBXA2R is shown in black, associated nodes are represented in blue, primary interactors of TBXA2 are shown in red and secondary interactors are shown in grey. The nodes are linked through physical interactions (data obtained from geneMania database). Abbreviations- ADRB1: Adrenoceptor Beta 1; ARRB2: Arrestin Beta 2; ADRB1: Arrestin Beta 1;AGTR1: Angiotensin II Receptor Type 1; AGTR1: Angiotensin II Receptor Type 1; ADRB2: Adrenoceptor Beta 2; TBXA2R: Thromboxane A2 Receptor; AGTRAP: Angiotensin II Receptor Associated Protein;BDKRB2: Bradykinin Receptor B2;CAV3:Caveolin 3; CDH15: Cadherin 15; CDH2:Cadherin 2;EDNRA: Endothelin Receptor Type A;G3BP2: G3BP Stress Granule Assembly Factor 2; GNA11: G Protein Subunit Alpha 11;GNAI2: G Protein Subunit Alpha I2;GNA12: G Protein Subunit Alpha 12;GNA13: G Protein Subunit Alpha 13;GNAQ: G Protein Subunit Alpha Q;GNAS: GNAS Complex Locus;GPRASP1: G Protein-Coupled Receptor Associated Sorting Protein 1;GRK5: G Protein-Coupled Receptor Kinase 5;HTR2B: 5-Hydroxytryptamine Receptor 2B;HRH2: Histamine Receptor H2; ITGB1BP1: Integrin Subunit Beta 1 Binding Protein 1; KCNMA1: Potassium Calcium-Activated Channel Subfamily M Alpha 1;NME1-MNE2: NME/NM23 nucleoside diphosphate kinase 1;OPRD1:Opioid Receptor Delta 1;OPRK1: Opioid Receptor Kappa 1;OPRM1: Opioid Receptor Mu 1;PRKCA: Protein Kinase C Alpha; RAF1: Raf-1 Proto-Oncogene, Serine/Threonine Kinase;WDR36: WD Repeat Domain 36; YWHAZ: Tyrosine 3-Monooxygenase/Tryptophan 5-Monooxygenase Activation Protein Zeta.

**Fig 4:**
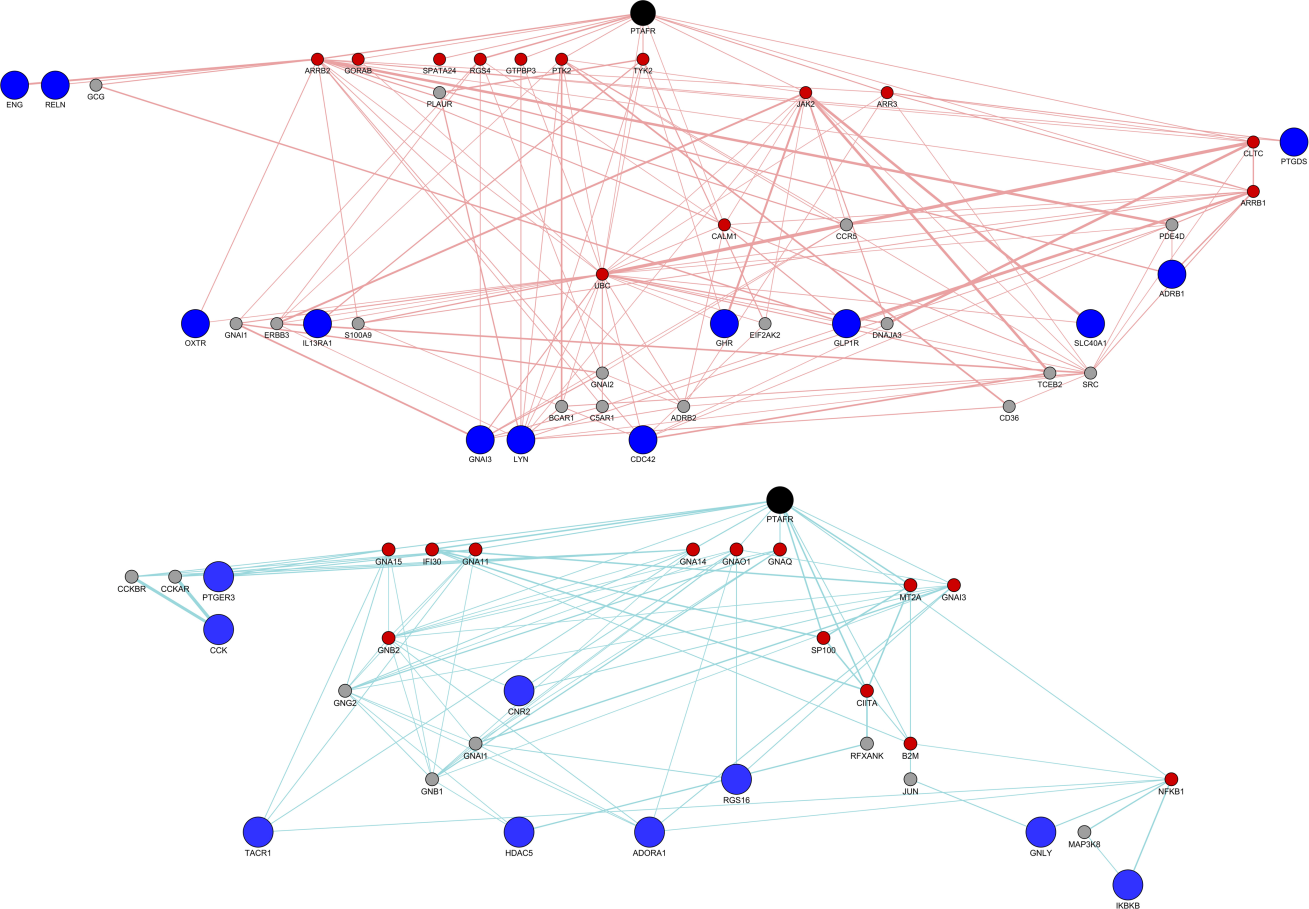
Clusters identified for PTAFR. PTAFR is shown in black, associated nodes from Table 1 are represented in blue, primary interactors of TBXA2R are shown in red and secondary interactors are shown in grey.The nodes are linked through physical interactions (red edges) and pathway interactions (blue edges). The data was obtained from geneMania software. Abbreviations- ADORA1: Adenosine A1 Receptor;ADRB1:Adrenoceptor Beta 1; CCK: Cholecystokinin; CDC42:Cell Division Cycle 42; CNR2:Cannabinoid Receptor 2;ENG:Endoglin;GHR:Growth Hormone Receptor; GLP1R: Glucagon Like Peptide 1 Receptor;GNAI3:G Protein Subunit Alpha I3;GNLY:Granulysin; HDAC5: Histone Deacetylase 5; IKBKB: Inhibitor Of Nuclear Factor Kappa B Kinase Subunit Beta; IL13RA1: Interleukin 13 Receptor Subunit Alpha 1; LYN:LYN Proto-Oncogene, Src Family Tyrosine Kinase; OXTR:Oxytocin Receptor;PTGDS: Prostaglandin D2 Synthase; PTAFR: platelet activating factor receptor;PTGER3: Prostaglandin E Receptor 3;RELN:Reelin; RGS16: Regulator Of G-Protein Signaling 16; SLC40A1: Solute Carrier Family 40 Member 1; TACR1: Tachykinin Receptor 1.

**Fig 5:**
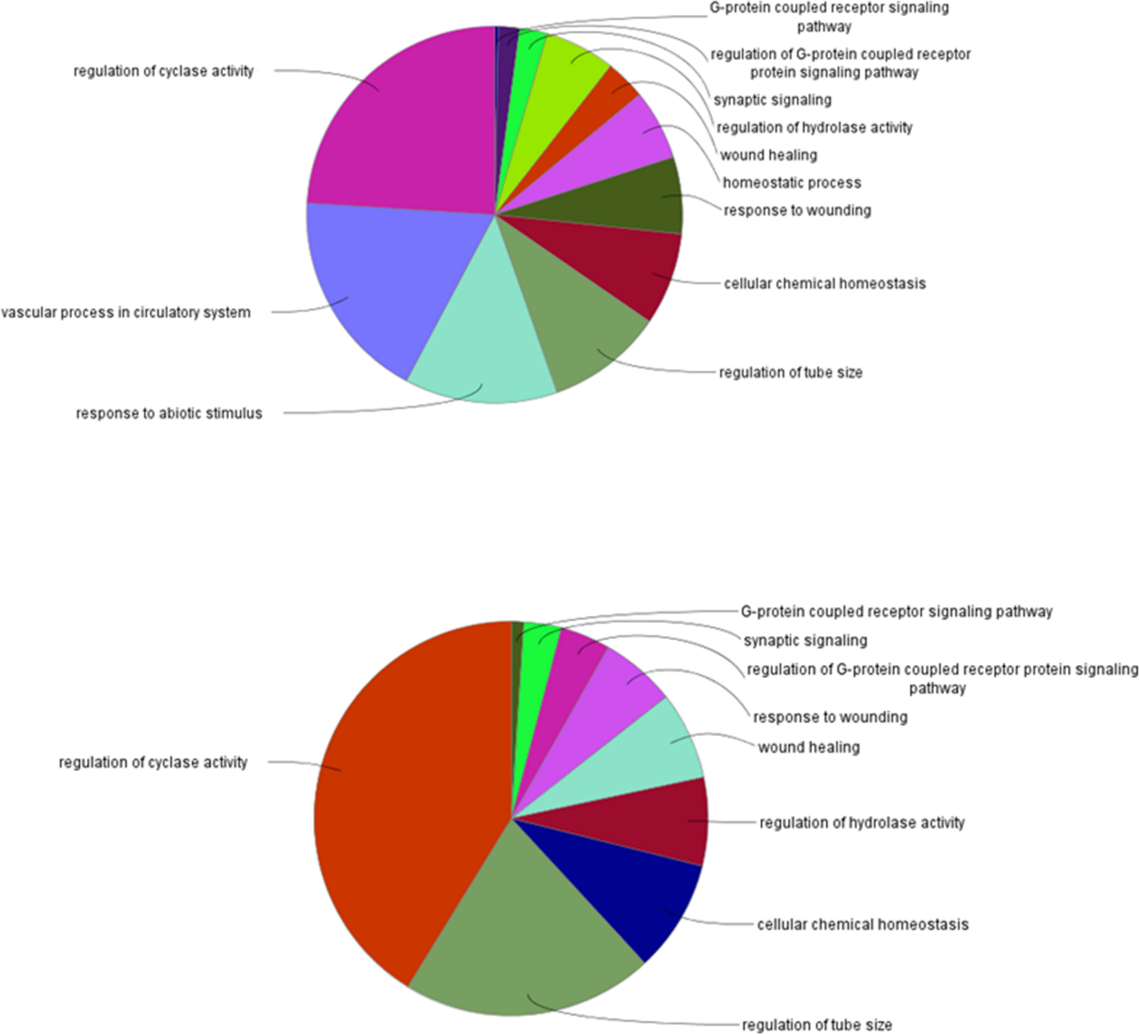
Biological processes involved in AP-associated pneumonia related to TBXA2R (upper panel) and PTAFR (lower panel). Abbreviation- TBXA2R: thromboxane A2 receptor; PTAFR:platelet activating factor receptor.

Findings on the potential role of TBXA2R and PTAFR in the increased risk of antipsychotic-associated pneumonia derived from CT-link were further investigated using Cytoscape. Several candidate genes encoding for proteins that physically interact with TBXA2R and PTAFR were identified. The pathways involved in the function of the clustered genes are shown in Figs 3 and 4. The identified AP off-targets, TBXA2R and PTAFR, in black, are linked, either directly or indirectly, to a group of nodes, shown in blue, which likely contribute to the increased risk of developing pneumonia-related symptoms after activation of TBXA2Ror PTAFR. The molecular cascade highlighted in these analyses is involved in a wide range of biological functions. The biological processes (identified from the Gene Ontology Database - http://geneontology.org/) related to the molecular cascade for both TBXA2R and PTAFR are indicated in Fig 5, while S3 and S4 Tables report the details of biological processes and sub-processes indicated in Fig 5. As shown by the supplementary files (S3–S6 Figs), several pathways were influenced by the function of the clustered nodes. While some of these clusters point to biological functions which seem completely unrelated to pneumonia-related symptoms, others are potentially related to pneumonia. Further detail on the web of biological functions related to PTAFR and TBAX2R are shown in the supplementary files (S7–S9 Figs).

## Discussion

Drug safety is an important public health concern, as can be seen by the continuous efforts in pharmacovigilance to identify, quantify and minimize drug-related risks. Over the years, antipsychotic safety has been investigated using both spontaneous reporting system databases as well as electronic medical records or claims databases. Whenever possible, the plausibility of associations between adverse drug events and drug use is strengthened by confirming the biological plausibility of such events, especially in case of “type A” adverse drug reaction which are dose-dependent and are based on pharmacological properties of the drug mechanism of action effects.

Of the 41 articles identified in the systematic literature review, only 30 hypothesized a pharmacological mechanism that could explain the antipsychotic-associated risk of pneumonia. Furthermore, only one of these studies was designed in such a way that could reasonably associate pneumonia to specific antipsychotic sub-groups, and therefore to a group of antipsychotics with closely related physicochemical properties (e.g., butyrophenones and phenothiazines among conventional antipsychotics)[4].

The approach underlying currently hypothesized mechanisms is limited because it is based on the traditional view of ‘systems pharmacology’, thus excluding a priori any“off-target” receptors and focusing on commonly known pharmacological systems, such as cholinergic, histaminergic, serotonergic and dopaminergic classes. As a result, other targets potentially mediating events of interest are not captured. A case in point is the identification of two novel receptor targets which were not identifiable based on the major pharmacologic systems.

The main clinical implications of the study findings primarily illustrate that antipsychotics of both classes have plausible mechanisms leading to pneumonia and/or lead to signs/symptoms that increase the risk of pneumonia. As a result, all APs should be used with caution and sparingly in persons at risk of pneumonia, especially the elderly. Indeed, the finding of similar risk of pneumonia across classes in the literature review conducted is consistent with a recently published systematic review and meta-analysis [44]. Nevertheless, the very diverse target binding among single APs, as highlighted by the analysis of experimentally-known AP target-binding, suggests that grouping antipsychotics into broad groups such as ‘atypical’ and ‘conventional’ may not be helpful or accurate. This method of classifying antipsychotics is based on the point in time when antipsychotics were marketed, with the older APs being termed conventional or first generation and newer ones being referred to as atypical or second generation. In vitro findings presented in this study confirm that such a classification is misleading, particularly concerning atypical APs, because there is a significant pharmacological heterogeneity among individual APs. Equally prone to being misleading are proposed mechanisms of action for pneumonia, or other adverse outcomes, that assumed all AP drugs to have the same or similar pharmacological action. The literature review conducted contains several such examples of mechanisms of action which related to all APs. Researchers should not be content with evaluating the risk of an outcome but should question the reason why such events occur, considering that biological feasibility is one of the key criteria for causality. The present study suggests that off-target pharmacology can play an important role in substantiating known signals. The novel mechanisms concerning TBXA2R and PTAFR as potential mediators of pneumonia symptoms constitute an important finding of this study.

PTAFR has already been linked to an increased risk of pneumococcal disease in particular by facilitating the adhesion of pneumococcal bacteria to endothelial cells in the respiratory tract, according to a recently published narrative review on the link between PTAFR and pneumococcal disease using in vitro and in vivodata[60]. Platelet-activating factor binds to PTAFR, activating the enzyme GTPase, up-regulating phospholipase C, D and A2 pathways and activating protein kinase C as well as tyrosine kinases, leading to an overall increase in inflammatory response[60]. TBXA2R appears to have been less extensively studied than PTAFR in the context of pneumonia, but has nevertheless been implicated in bronchial hyper-responsiveness [61] as well as bronchial constriction [62] in animal models, both of which may be risk factors for pneumonia. We hypothesize that the increased risk of pneumonia-like symptoms mediated by PTAFR and TBXA2R may be exerted through the control of capillary permeability at the alveolar level. Impaired control of permeability may increase the quantity of fluids in the alveoli, thus causing edema, a primary risk factor as well as symptom of pneumonia. In addition, pathways involved with the gene/protein clusters identified in relation to pneumonia symptoms also have a role in maintaining the structure of blood vessels. Impairment of this function may negatively impact circulatory system’s physical properties, leading to leaking of fluids in the alveoli. Control of platelet and coagulation homeostasis may also play a role in the increase of fluids within alveoli after TBXA2R and PTAFR activation. Future studies should aim to quantify the affinity of APs and other drugs associated with a risk of pneumonia also to these two receptors in vitro. From a clinical point of view, it is relevant to identify the drug receptor-binding profiles related leading to a specific adverse effect, in this case, pneumonia, as this may help prevent the concomitant use of drugs having a potentially synergistic or additive risk of that adverse effect. Furthermore, future studies should focus more on the single drug safety profile rather than grouping antipsychotic drugs by class, as this may help to identify high risk drugs as well as high risk patient groups and better support individualized medicine.

This paper has several strengths. The literature review allowed us to screen published material for hypothesized mechanisms underlying pneumonia and understand what importance is given to this aspect of drug safety. In addition, the in silicoapproaches to drug safety substantiation used in the present paper build on each other, first using off-target pharmacology to identify relevant novel receptors associated with pneumonia and then using Cytoscape to construct likely biological cascades connecting the novel receptors to pneumonia symptoms. Indeed, similar approaches to better drug safety, such as “medication-wide association studies”[63] and systems pharmacology [64] have been proposed to augment the capacity of currently used pharmacovigilance systems. Findings from this study confirmed existing receptor targets for AP drugs but also provided two novel off-targets that may lead to AP-associated pneumonia, namely PTAFR and TBXA2R.

There are however some limitations that should be borne in mind. Some of the hypothesized signal substantiation mechanisms suggested in the published literature were found to be of limited value as they did not mention a specific drug but focused on an entire drug class (conventional or atypical) or even on antipsychotics in general. This constituted a limitation in the starting point of collecting hypotheses, as it is unlikely that such suggested mechanisms underlying AP-associated pneumonia are completely reliable given the physicochemical differences between each drug. With regards to the safety predictions generated by CT-link and the generation of molecular cascades with Cytoscape, there are at least two inherent limitations associated with any approach relying on signals therein. On one hand, the experimentally known in vitro affinity data for drugs may be incomplete [14], which can have an impact on the list of proteins included in the target signatures linked to safety terms and thus, on the ability to identify protein targets likely to be responsible for the AP-associated pneumonia. Given the data sources used to carry out this study, it was not possible to examine whether there are some mechanisms that are more strongly associated with the occurrence of pneumonia than others. Finally, this work focused on a set of seven antipsychotic drugs for which there is a considerable amount of bibliographical information in the context of the risk of pneumonia [25–29].Therefore, our results cannot be considered exhaustive with regards to the current portfolio of over 50 antipsychotic medications but can certainly be taken as a reference from which substantiation analyses of AP-associated pneumonia could be performed.

## Conclusion

The literature review identified several biological mechanisms that could lead to pneumonia with AP use. In vitro pharmacology data confirmed receptor affinities identified in theliterature review. Two targets, thromboxane A2 receptor (TBXA2R) and plateletactivating factor receptor (PTAFR) were found to be novel AP target receptorspotentially associated with pneumonia. Biological pathways constructed usingCytoscape identified plausible biological links potentially leading to pneumoniadownstream of TBXA2R and PTAFR. Innovative approaches for biologicalsubstantiation of drug-adverse event associations may strengthen evidence on drugsafety profiles and help tailoring pharmacological therapies.

## Supporting information

S1 Fig PRISMA checklist. (PDF)

S2 Fig Detail of study extraction during systematic review. Abbreviations- AP: antipsychotic; RCT: randomized controlled trial.
(TIFF)

S3 Fig Flowchart of the analysis carried out in Cytoscape. Abbreviations- ANs: associated nodes; AP: antipsychotic. Definitions- Biological net: This refers to a group of genes/proteins which interact with each other. Associated nodes: The term refers to genes/proteins, within the biological net, which are linked to pneumonia related processes, according to literature data (mainly from Medline database).
(TIFF)

S4 Fig **Panel A: The biological net associated with TBXA2R obtained through Cytoscape’s “Physical Interaction” enrichment. Panel B: The direct interactors of TBXA2R within the biological net. Panel C: the downstream elements of the biological net (in blue) and the connecting nodes (in grey).**Abbreviation-TBXA2R: thromboxane A2 receptor.
(TIFF)

S5 Fig **Panel A: The biological net associated with PTAFR obtained through Cytoscape’s “Physical Interaction” enrichment. Panel B: The direct interactors of PTAFR within the biological net. Panel C: the downstream elements of the biological net (in blue) and the connecting nodes (in grey).**Abbreviation- PTAFR: platelet activating factor receptor.
(TIFF)

S6 Fig **Panel A: The biological net related to TBXA2R obtained through Cytoscape’s “Pathway interaction” enrichment. Panel B: Direct interactors of TBXA2R within the biological net. Panel C: The downstream elements of the biological net (in blue) and the connecting nodes (in grey).**Abbreviation-TBXA2R: thromboxane A2 receptor.
(TIFF)

S7 Fig **Panel A: the biological net related to PTAFR obtained through Cytoscape’s “pathway interaction” enrichment. Panel B: direct interactors of PTAFR within the biological net. Panel B: the downstream elements of the biological net (in blue) and the connecting nodes (in grey).**Abbreviation- PTAFR: platelet activating factor receptor.
(TIFF)

S8 Fig Biological processes (colored captions and nodes) and sub-processes (captions underneath each node) associated with TBXA2R-related elements. Nodes belonging to multiple processes are indicated by multicolored sections.Abbreviation-TBXA2R: thromboxane A2 receptor.
(TIFF)

S9 Fig Biological processes (colored captions and nodes) and sub-processes (captions underneath each node) associated with PTAFR-related elements. Nodes belonging to multiple processes are indicated by multicolored sections. Abbreviation- PTAFR: platelet activating factor receptor.
(TIFF)

S1 Table List of studies excluded due to lack of available full-text. The information in table above was identified from the abstract, where this was available.
(DOCX)

S2 Table Main respiratory safety terms, potentially related to pneumonia, which may be associated with antipsychotics as found in CT-link. (DOCX)

S3 Table The biological processes associated with the genes of biological net obtained for TBXA2R. Abbreviation-TBXA2R: thromboxane A2 receptor.
(DOCX)

S4 Table The biological processes associated with the genes of biological net obtained for PTAFR. Abbreviation- PTAFR: platelet activating factor receptor.
(DOCX)
