# Supplementary figures and images for: Biological substantiation of antipsychotic-associated pneumonia: Systematic literature review and computational analyses
Source: PLoS One. 2017 Oct 27;12(10):e0187034. doi: 10.1371/journal.pone.0187034 (PMC5659779; doi:10.1371/journal.pone.0187034)

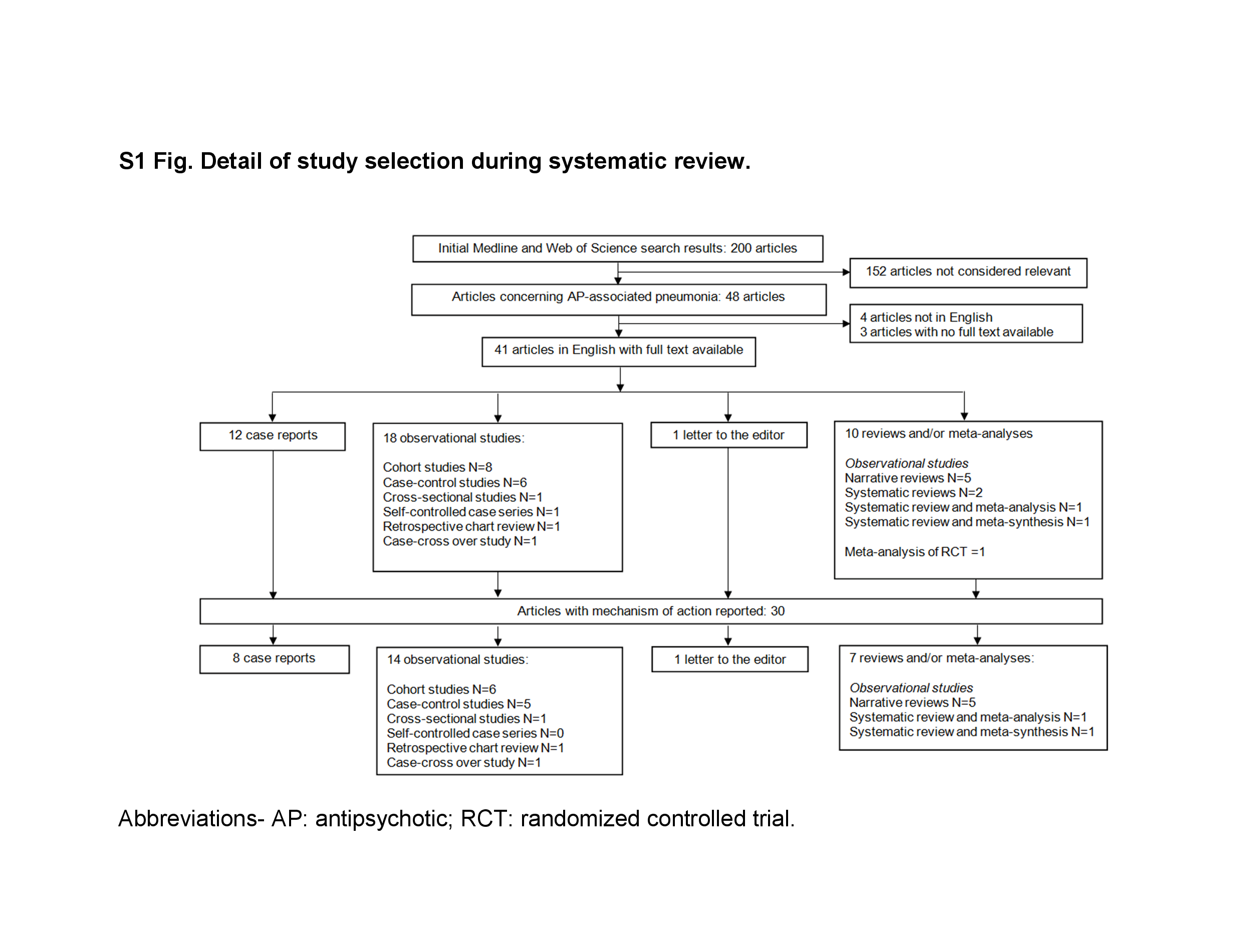

Supplement: S2 Fig — Abbreviations- AP: antipsychotic; RCT: randomized controlled trial. (TIFF) [file pone.0187034.s002.tiff]

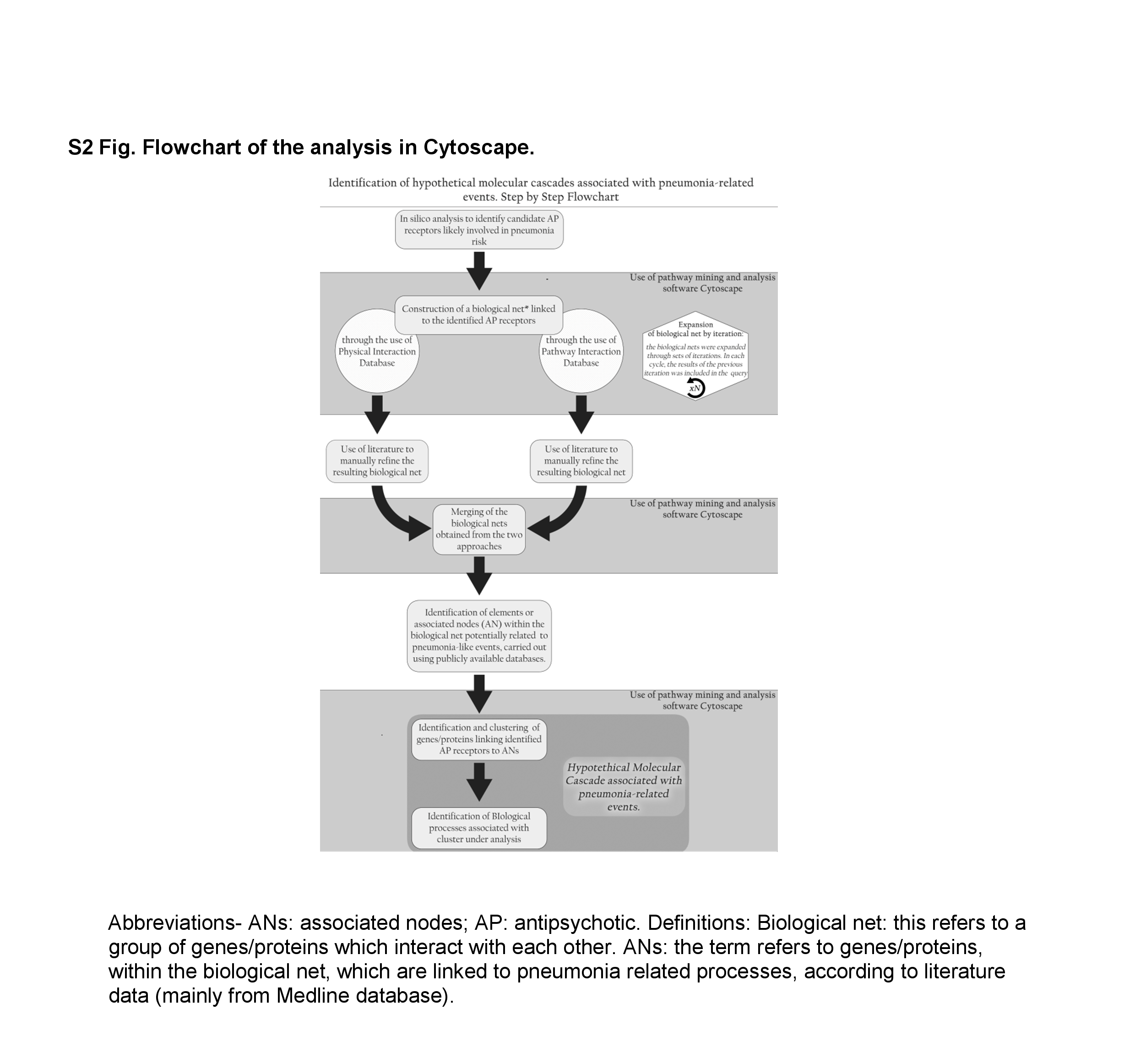

Supplement: S3 Fig — Abbreviations- ANs: associated nodes; AP: antipsychotic. Definitions- Biological net: This refers to a group of genes/proteins which interact with each other. Associated nodes: The term refers to genes/proteins, within the biological net, which are linked to pneumonia related processes, according to literature data (mainly from Medline database). (TIFF) [file pone.0187034.s003.tiff]

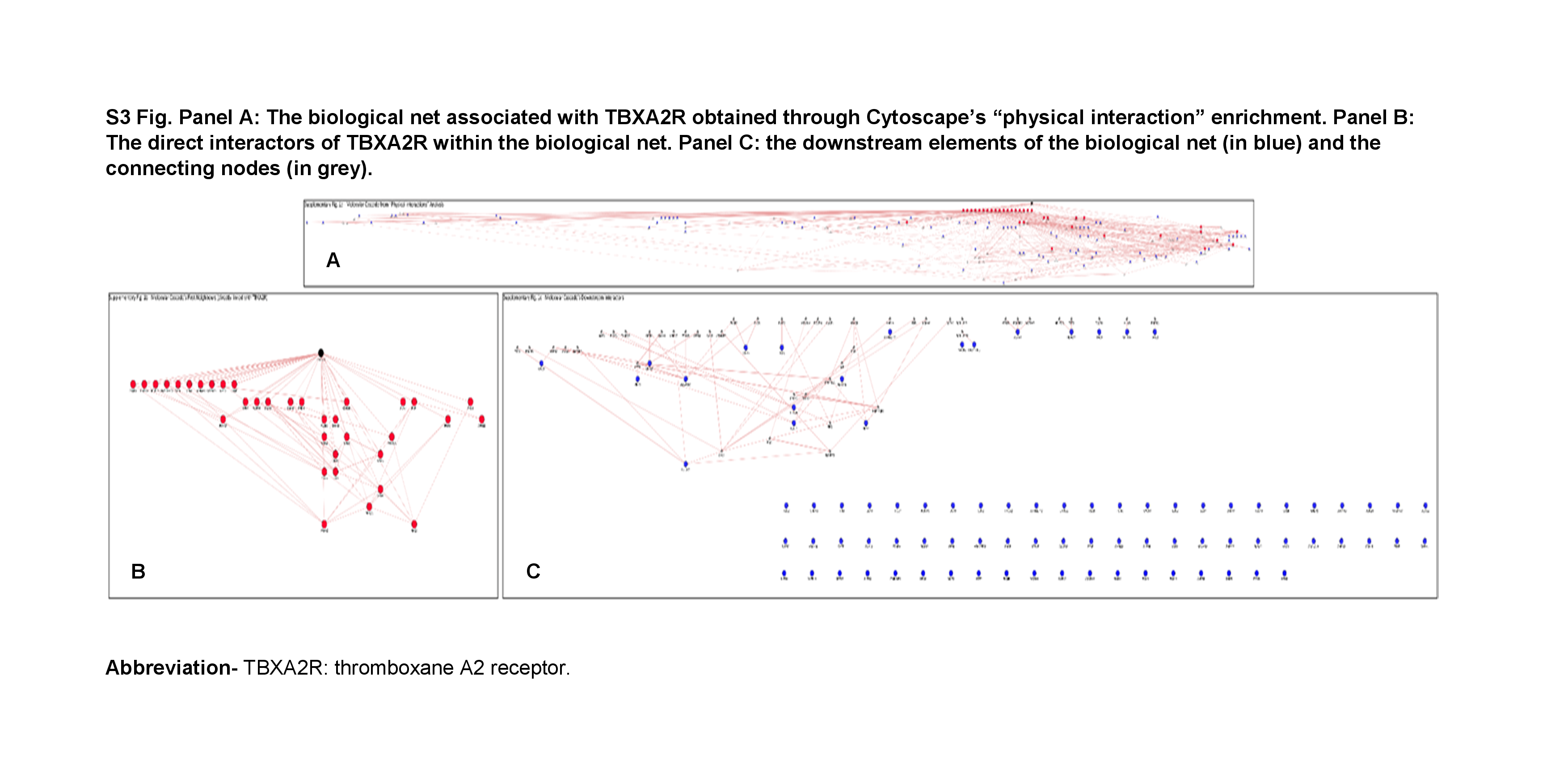

Supplement: S4 Fig — Panel A: The biological net associated with TBXA2R obtained through Cytoscape’s “Physical Interaction” enrichment. Panel B: The direct interactors of TBXA2R within the biological net. Panel C: the downstream elements of the biological net (in blue) and the connecting nodes (in grey).Abbreviation-TBXA2R: thromboxane A2 receptor. (TIFF) [file pone.0187034.s004.tiff]

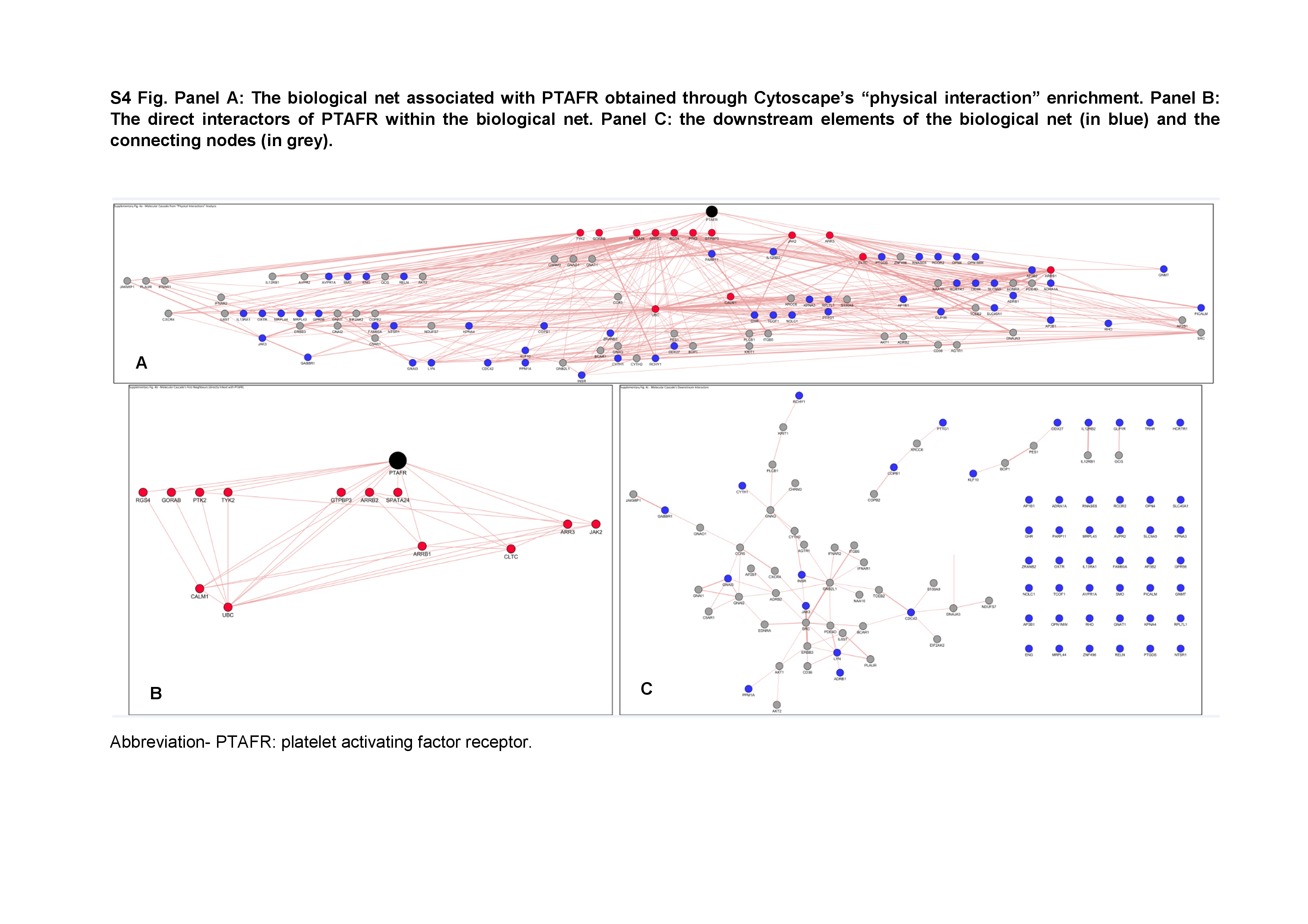

Supplement: S5 Fig — Panel A: The biological net associated with PTAFR obtained through Cytoscape’s “Physical Interaction” enrichment. Panel B: The direct interactors of PTAFR within the biological net. Panel C: the downstream elements of the biological net (in blue) and the connecting nodes (in grey).Abbreviation- PTAFR: platelet activating factor receptor. (TIFF) [file pone.0187034.s005.tiff]

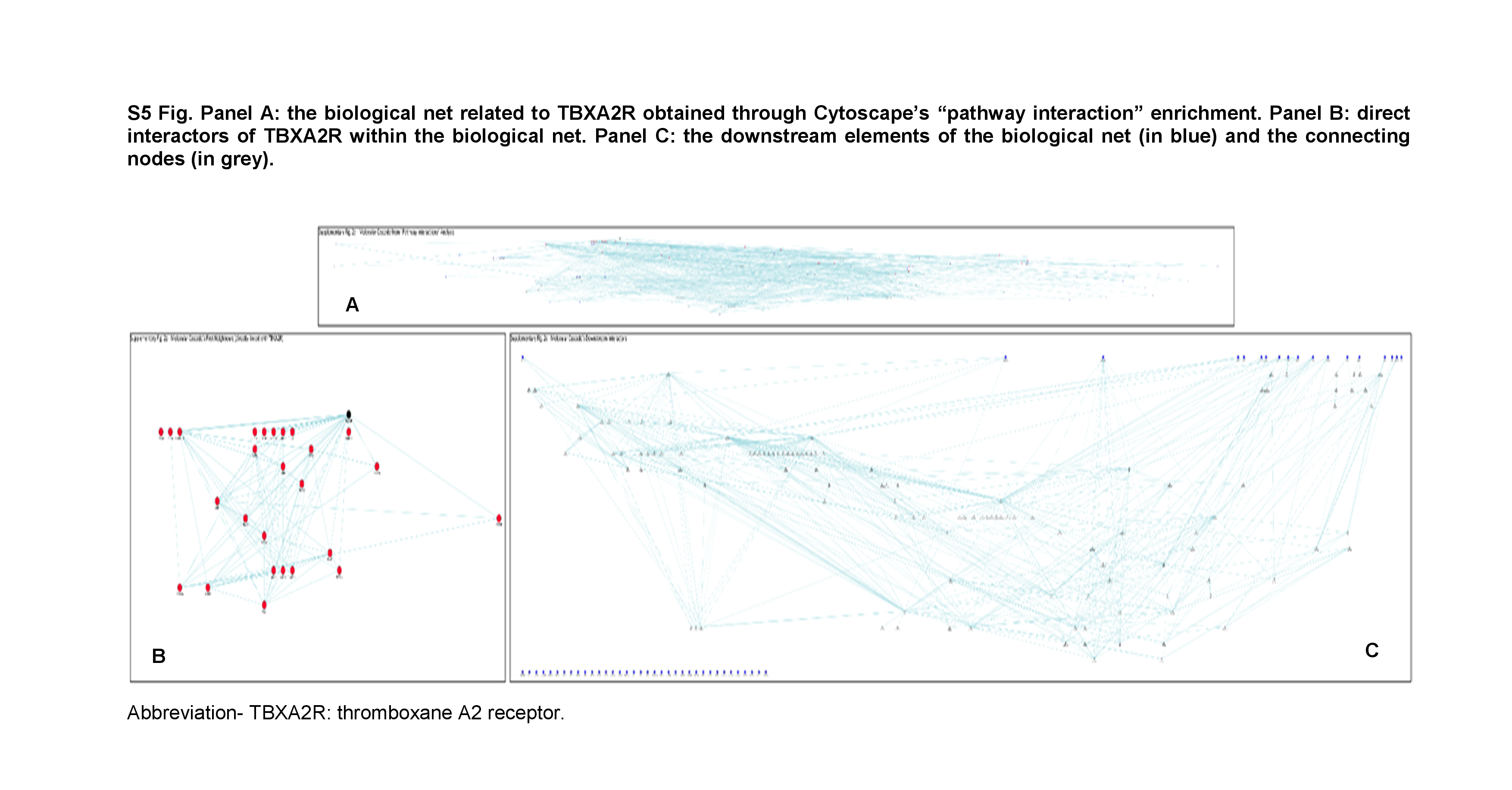

Supplement: S6 Fig — Panel A: The biological net related to TBXA2R obtained through Cytoscape’s “Pathway interaction” enrichment. Panel B: Direct interactors of TBXA2R within the biological net. Panel C: The downstream elements of the biological net (in blue) and the connecting nodes (in grey).Abbreviation-TBXA2R: thromboxane A2 receptor. (TIFF) [file pone.0187034.s006.tiff]

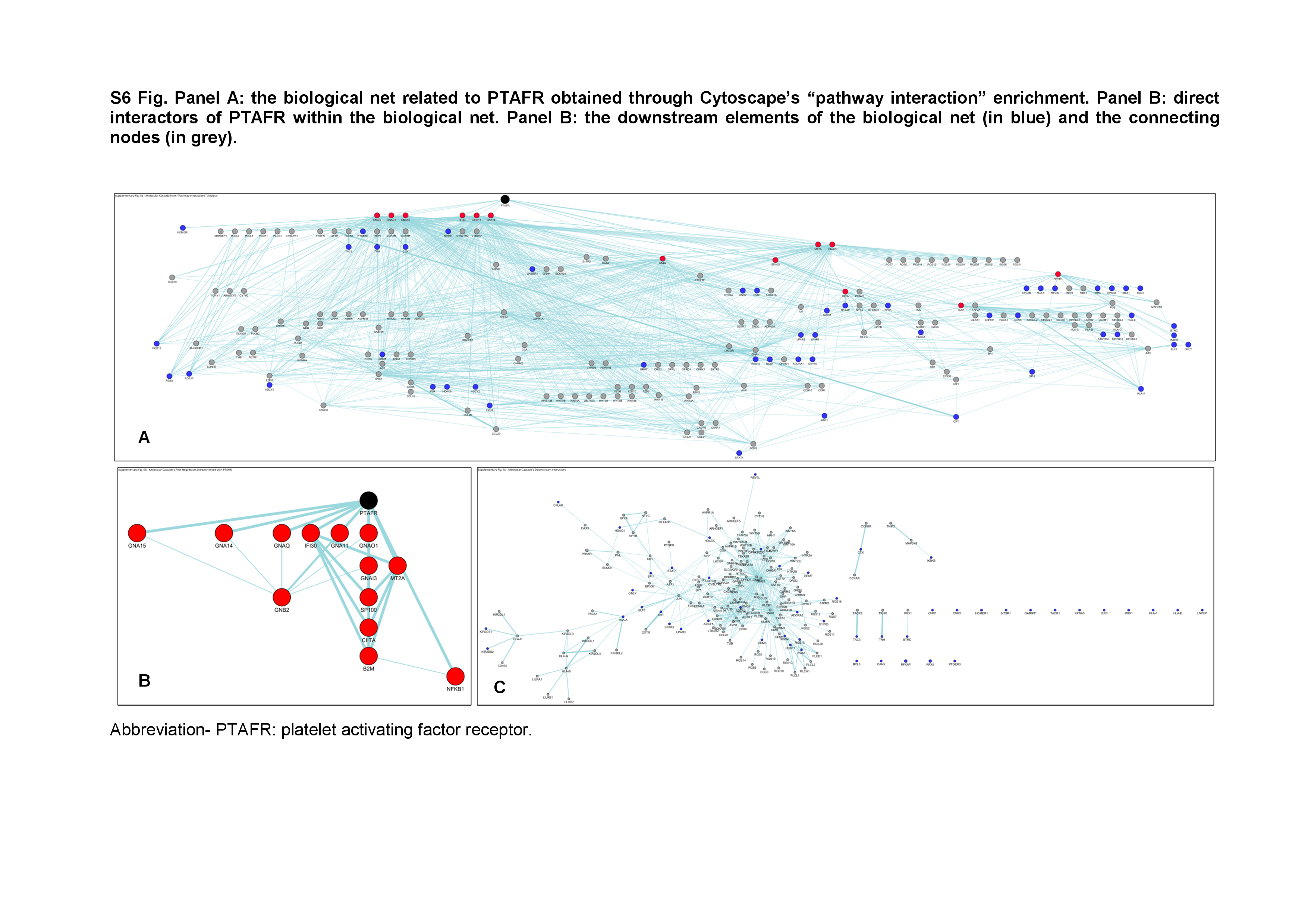

Supplement: S7 Fig — Panel A: the biological net related to PTAFR obtained through Cytoscape’s “pathway interaction” enrichment. Panel B: direct interactors of PTAFR within the biological net. Panel B: the downstream elements of the biological net (in blue) and the connecting nodes (in grey).Abbreviation- PTAFR: platelet activating factor receptor. (TIFF) [file pone.0187034.s007.tiff]

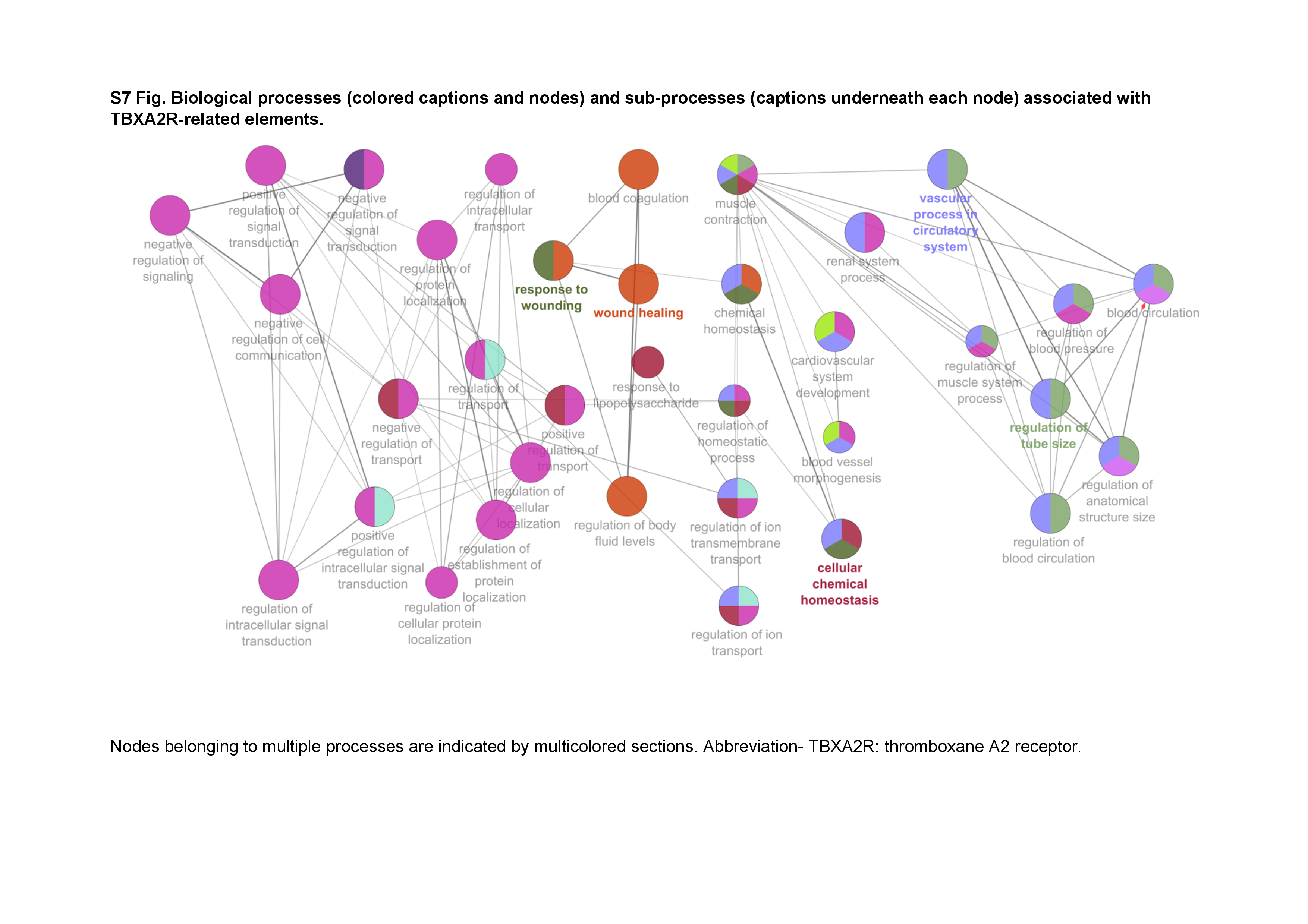

Supplement: S8 Fig — Nodes belonging to multiple processes are indicated by multicolored sections.Abbreviation-TBXA2R: thromboxane A2 receptor. (TIFF) [file pone.0187034.s008.tiff]

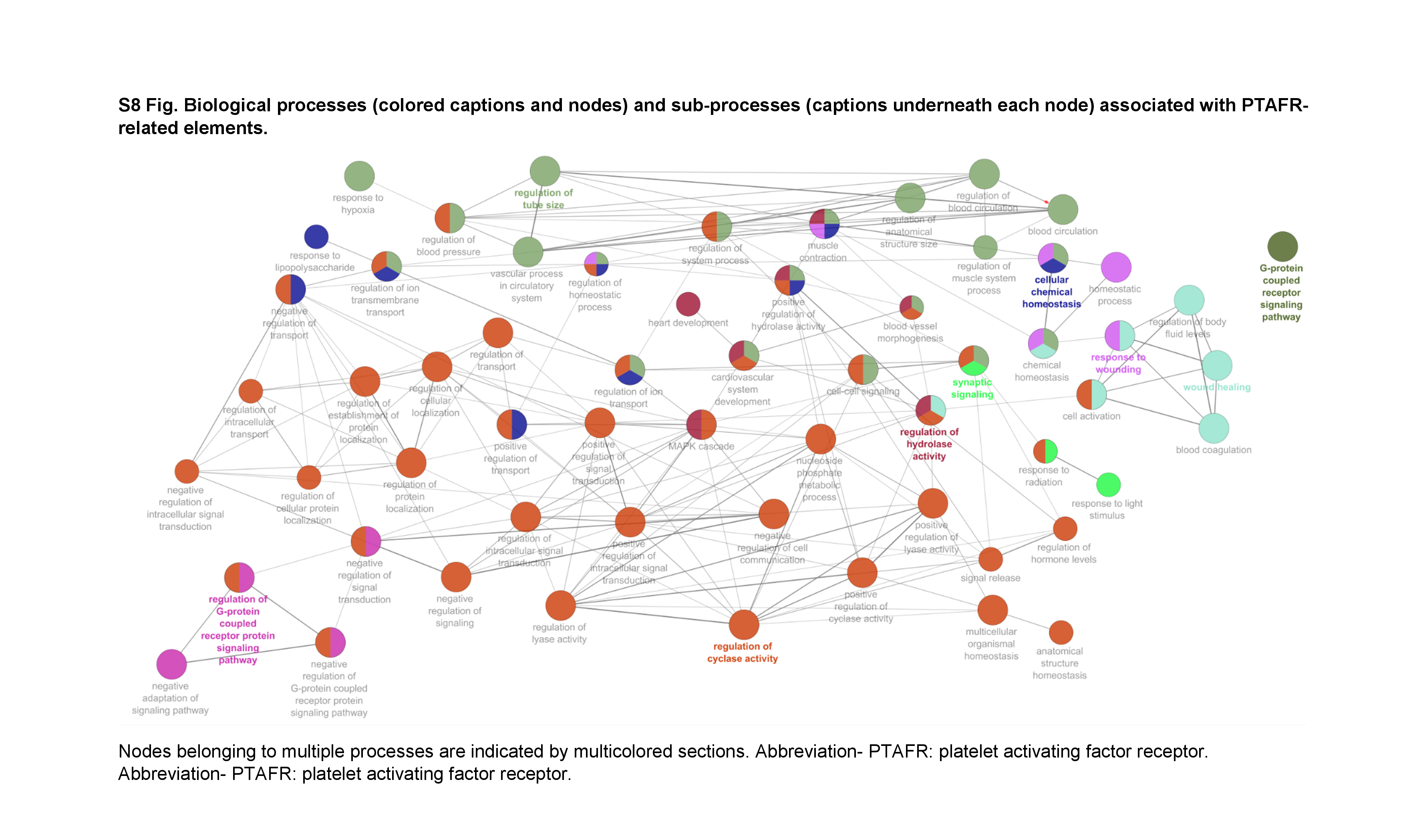

Supplement: S9 Fig — Nodes belonging to multiple processes are indicated by multicolored sections. Abbreviation- PTAFR: platelet activating factor receptor. (TIFF) [file pone.0187034.s009.tiff]
