# Supplementary material for: Biological substantiation of antipsychotic-associated pneumonia: Systematic literature review and computational analyses
Source: PLoS One. 2017 Oct 27;12(10):e0187034. doi: 10.1371/journal.pone.0187034 (PMC5659779; doi:10.1371/journal.pone.0187034)
Supplement: S1 Table — The information in table above was identified from the abstract, where this was available. (DOCX) [file pone.0187034.s010.docx]

**S1 Table. List of studies excluded due to lack of available full-text, even after authors were contacted for a copy of the article.**

| **Type of study** | **Study population/patient** | **Exposure** | **Mechanisms hypothesized** | **Outcome and/or risk estimate** |
| --- | --- | --- | --- | --- |
| Case report [[1](#_ENREF_1)] | No information in abstract | Clozapine | Severe clozapine-induced constipation leading to dysfunction of the diaphragm, leading to pneumonia | Pneumonia |
| Comment to the editor [[2](#_ENREF_2)] | No abstract available | | | |
| Case report [[3](#_ENREF_3)] | 74-year old female | Trifluoperazine | Drug-induced Parkinsonism, leading to dysphagia, leading to aspiration pneumonia | Aspiration pneumonia |

The information in the table above was identified from the abstract where this was available.

**References**

1. Galappathie N,S Khan, (2014): *Clozapine-associated pneumonia and respiratory arrest secondary to severe constipation.* Med Sci Law, 54(2): p. 105-9.

2. Haddad PM, (2013): *Current use of second-generation antipsychotics may increase risk of pneumonia in people with schizophrenia.* Evid Based Ment Health, **16**(4): p. 109.

3. Bashford G,P Bradd, (1996): *Drug-induced Parkinsonism associated with dysphagia and aspiration: a brief report.* J Geriatr Psychiatry Neurol, **9**(3): p. 133-5.
