# Supplementary material for: Biological substantiation of antipsychotic-associated pneumonia: Systematic literature review and computational analyses
Source: PLoS One. 2017 Oct 27;12(10):e0187034. doi: 10.1371/journal.pone.0187034 (PMC5659779; doi:10.1371/journal.pone.0187034)
Supplement: S2 Table — (DOCX) [file pone.0187034.s011.docx]

**S2 Table. Main respiratory safety terms, potentially related to pneumonia, which may be associated with antipsychotics as found in CT-link.**

|  | **Associated antipsychotic drugs** | |
| --- | --- | --- |
| **Safety term** | **Known associations** | **Predicted associations** |
| Airway obstruction | Amisulpride, haloperidol, quetiapine | - |
| Alveolitis | Haloperidol, quetiapine | Amisulpride, clozapine, risperidone, zotepine, olanzapine |
| Asphyxia | Amisulpride, haloperidol, quetiapine, risperidone | - |
| Cardiorespiratory failure | Amisulpride, haloperidol, quetiapine, risperidone, zotepine | - |
| Dyspnea | Amisulpride, haloperidol, quetiapine, risperidone | Clozapine, zotepine |
| Hypoxia | Quetiapine, risperidone | - |
| Lung diseases | Amisulpride, haloperidol, quetiapine, risperidone, zotepine | Clozapine, olanzapine |
| Pulmonary edema | Amisulpride, haloperidol, risperidone, | Zotepine |
| Respiration disorders | Amisulpride, haloperidol, quetiapine, risperidone, zotepine | Clozapine |
| Respiratory distress | Haloperidol, quetiapine | - |
| Slow respiration | Quetiapine, risperidone | - |
