# Supplementary material for: Biological substantiation of antipsychotic-associated pneumonia: Systematic literature review and computational analyses
Source: PLoS One. 2017 Oct 27;12(10):e0187034. doi: 10.1371/journal.pone.0187034 (PMC5659779; doi:10.1371/journal.pone.0187034)
Supplement: S4 Table — Abbreviation- PTAFR: platelet activating factor receptor. (DOCX) [file pone.0187034.s013.docx]

**S4 Table. The biological processes associated with the genes of biological net obtained for PTAFR.**

| **Cluster** | **Terms within cluster** | **Genes associated with each term** |
| --- | --- | --- |
| Regulation of tube size | Regulation of homeostatic processes | *AGTR1, CAV3, EDNRA, HTR2B, OPRK1, PRKCA, RACK1* |
|  | Regulation of anatomical structure size | *ADRB1, ADRB2, AGTR1, BDKRB2, CAV3, EDNRA, HRH2, HTR2B, ITGB1BP1, KCNMA1, TBXA2R, WDR36* |
|  | Cell-cell signalling | *ADRB1, ADRB2, AGTR1, ARRB2, GNAI2, GNAS, GNB1, HRH2, KCNMA1, OPRD1, OPRK1, OPRM1, PRKCA, RAF1* |
|  | Regulation of system process | *ADRB1, ADRB2, AGTR1, BDKRB2, CAV3, HRH2, KCNMA1, OPRD1, OPRK1, OPRM1, PRKCA, TBXA2R* |
|  | Response to hypoxia | *ADRB2, AGTRAP, BDKRB2, EDNRA, GNB1, KCNMA1, OPRD1, RAF1* |
|  | Vascular process in circulatory system | *ADRB1, ADRB2, AGTR1, BDKRB2, EDNRA, HRH2, HTR2B, ITGB1BP1, KCNMA1, TBXA2R* |
|  | Muscle contraction | *ADRB2, BDKRB2, CAV3, EDNRA, HTR2B, KCNMA1, PRKCA, TBXA2R* |
|  | Blood circulation | *ADRB1, ADRB2, AGTR1, AGTRAP, BDKRB2, CAV3, EDNRA, HRH2, HTR2B, ITGB1BP1, KCNMA1, PRKCA, TBXA2R* |
|  | Regulation of blood pressure | *ADRB1, ADRB2, AGTR1, AGTRAP, BDKRB2, EDNRA, TBXA2R* |
|  | Regulation of tube size | *ADRB1, ADRB2, AGTR1, BDKRB2, EDNRA, HRH2, HTR2B, ITGB1BP1, KCNMA1, TBXA2R* |
|  | Chemical homeostasis | *AGTR1, BDKRB2, CAV3, EDNRA, GNA13, GNAI2, GNAS, GNB1, HTR2B, KCNMA1, OPRM1, PRKCA, RACK1, RAF1, TBXA2R* |
|  | Cellular chemical homeostasis | *AGTR1, BDKRB2, CAV3, EDNRA, GNA13, GNB1, HTR2B, KCNMA1, OPRM1, PRKCA, RACK1, RAF1, TBXA2R* |
|  | Regulation of muscle system process | *ADRB2, CAV3, KCNMA1, PRKCA, TBXA2R* |
|  | Synaptic signalling | *ADRB2, ARRB2, GNAI2, GNB1, HRH2, KCNMA1, OPRD1, OPRK1, OPRM1, PRKCA, RAF1* |
|  | Regulation of blood circulation | *ADRB1, ADRB2, AGTR1, BDKRB2, CAV3, HRH2, PRKCA, TBXA2R* |
|  | Regulation of ion transport | *ADRB2, ARRB2, CAV3, EDNRA, GNAI2, GRK6, KCNMA1, OPRD1, OPRK1, OPRM1, PRKCA* |
|  | Positive regulation of hydrolase activity | *ADRB1, ADRB2, AGTR1, EDNRA, GNA13, GNAQ, GNAS, HTR2B, PRKCA, RACK1, TBXA2R* |
|  | Cardiovascular system development | *ADRB2, CAV3, CDH2, EDNRA, GNA11, GNA13, GNAQ, HTR2B, ITGB1BP1, PRKCA, RAF1, TBXA2R* |
|  | Regulation of ion transmembrane transport | *CAV3, EDNRA, GRK6, KCNMA1, OPRK1, OPRM1, PRKCA* |
|  | Blood vessel morphogenesis | *ADRB2, CDH2, EDNRA, GNA13, ITGB1BP1, PRKCA, TBXA2R* |
| Synaptic signalling | Response to radiation | *GNA11, GNAQ, GNB1, HRH2, OPRK1, OPRM1, PRKCA* |
|  | Response to light stimulus | *GNA11, GNAQ, GNB1, HRH2, PRKCA* |
|  | Synaptic signalling | *ADRB2, ARRB2, GNAI2, GNB1, HRH2, KCNMA1, OPRD1, OPRK1, OPRM1, PRKCA, RAF1* |
| Wound healing | Cell activation | *ADRB2, ARRB2, GNA11, GNA12, GNA13, GNAI2, GNAQ, GNAS, GNB1, HTR2B, PRKCA, RAF1, TBXA2R, YWHAZ* |
|  | Response to wounding | *ADRB2, AGTR1, ARRB2, CAV3, CDH15, EDNRA, GNA11, GNA12, GNA13, GNAI2, GNAQ, GNAS, GNB1, KCNMA1, OPRM1, PRKCA, RAF1, TBXA2R, YWHAZ* |
|  | Regulation of body fluid levels | *ADRB2, ARRB2, GNA11, GNA12, GNA13, GNAI2, GNAQ, GNAS, GNB1, KCNMA1, OPRK1, PRKCA, RAF1, TBXA2R, YWHAZ* |
|  | Wound healing | *ADRB2, ARRB2, CAV3, CDH15, GNA11, GNA12, GNA13, GNAI2, GNAQ, GNAS, GNB1, KCNMA1, OPRM1, PRKCA, RAF1, TBXA2R, YWHAZ* |
|  | Chemical homeostasis | *AGTR1, BDKRB2, CAV3, EDNRA, GNA13, GNAI2, GNAS, GNB1, HTR2B, KCNMA1, OPRM1, PRKCA, RACK1, RAF1, TBXA2R* |
|  | Blood coagulation | *ADRB2, ARRB2, GNA11, GNA12, GNA13, GNAI2, GNAQ, GNAS, GNB1, KCNMA1, PRKCA, RAF1, TBXA2R, YWHAZ* |
|  | Regulation of hydrolase activity | *ADRB1, ADRB2, AGTR1, ARRB2, EDNRA, GNA13, GNAQ, GNAS, HTR2B, ITGB1BP1, PRKCA, RACK1, RAF1, TBXA2R* |
| Cellular chemical homeostasis | Regulation of homeostatic process | *AGTR1, CAV3, EDNRA, HTR2B, OPRK1, PRKCA, RACK1* |
|  | Positive regulation of transport | *ADRB2, ARRB2, CAV3, CDH15, EDNRA, GNAI2, HTR2B, ITGB1BP1, OPRK1, RACK1, YWHAZ* |
|  | Negative regulation of transport | *CAV3, G3BP2, GRK6, ITGB1BP1, OPRK1, OPRM1, PRKCA, RACK1* |
|  | Muscle contraction | *ADRB2, BDKRB2, CAV3, EDNRA, HTR2B, KCNMA1, PRKCA, TBXA2R* |
|  | Cellular chemical homeostasis | *AGTR1, BDKRB2, CAV3, EDNRA, GNA13, GNB1, HTR2B, KCNMA1, OPRM1, PRKCA, RACK1, RAF1, TBXA2R* |
|  | Regulation of ion transport | *ADRB2, ARRB2, CAV3, EDNRA, GNAI2, GRK6, KCNMA1, OPRD1, OPRK1, OPRM1, PRKCA* |
|  | Positive regulation of hydrolase activity | *ADRB1, ADRB2, AGTR1, EDNRA, GNA13, GNAQ, GNAS, HTR2B, PRKCA, RACK1, TBXA2R* |
|  | Response to lipopolysaccharide | *EDNRA, OPRK1, OPRM1, PRKCA, TBXA2R* |
|  | Regulation of ion transmembrane transport | *CAV3, EDNRA, GRK6, KCNMA1, OPRK1, OPRM1, PRKCA* |
| Regulation of G-protein coupled receptor protein signaling pathway | Negative regulation of signal transduction | *ADRB2, ARRB2, BDKRB2, CAV3, CDH2, G3BP2, GPRASP1, GRK5, GRK6, HTR2B, ITGB1BP1, OPRM1, PRKCA, RACK1, RAF1* |
|  | Regulation of G-protein coupled receptor protein signaling pathway | *ADRB2, ARRB2, GNB1, GPRASP1, GRK5, GRK6, HTR2B, PRKCA* |
|  | Negative adaptation of signaling pathway | *ADRB2, ARRB2, GRK5, GRK6, HTR2B* |
|  | Negative regulation of G-protein coupled receptor protein signaling pathway | *ADRB2, ARRB2, GPRASP1, GRK5, GRK6, HTR2B* |
| Regulation of cyclase activity | Cell activation | *ADRB2, ARRB2, GNA11, GNA12, GNA13, GNAI2, GNAQ, GNAS, GNB1, HTR2B, PRKCA, RAF1, TBXA2R, YWHAZ* |
|  | Response to radiation | *GNA11, GNAQ, GNB1, HRH2, OPRK1, OPRM1, PRKCA* |
|  | Regulation of hormone levels | *AGTR1, GNAI2, GNAS, KCNMA1, OPRK1, PRKCA, RAF1* |
|  | Negative regulation of signaling | *ADRB2, ARRB2, BDKRB2, CAV3, CDH2, G3BP2, GNAI2, GPRASP1, GRK5, GRK6, HTR2B, ITGB1BP1, OPRK1, OPRM1, PRKCA, RACK1, RAF1* |
|  | Regulation of homeostatic process | *AGTR1, CAV3, EDNRA, HTR2B, OPRK1, PRKCA, RACK1* |
|  | Cell-cell signaling | *ADRB1, ADRB2, AGTR1, ARRB2, GNAI2, GNAS, GNB1, HRH2, KCNMA1, OPRD1, OPRK1, OPRM1, PRKCA, RAF1* |
|  | Regulation of protein localization | *CAV3, CDH2, G3BP2, GNAI2, GNAQ, GNAS, HTR2B, ITGB1BP1, OPRM1, PRKCA, RACK1, YWHAZ* |
|  | Regulation of system process | *ADRB1, ADRB2, AGTR1, BDKRB2, CAV3, HRH2, KCNMA1, OPRD1, OPRK1, OPRM1, PRKCA, TBXA2R* |
|  | Multicellular organismal homeostasis | *ADRB1, ADRB2, CDH15, GNAS, OPRK1, PRKCA, WDR36* |
|  | Regulation of transport | *ADRB2, AGTR1, ARRB2, CAV3, CDH15, EDNRA, G3BP2, GNAI2, GNAQ, GNAS, GRK6, HTR2B, ITGB1BP1, KCNMA1, OPRD1, OPRK1, OPRM1, PRKCA, RACK1, YWHAZ* |
|  | Positive regulation of transport | *ADRB2, ARRB2, CAV3, CDH15, EDNRA, GNAI2, HTR2B, ITGB1BP1, OPRK1, RACK1, YWHAZ* |
|  | Negative regulation of transport | *CAV3, G3BP2, GRK6, ITGB1BP1, OPRK1, OPRM1, PRKCA, RACK1* |
|  | Regulation of cellular localization | *CAV3, CDH15, CDH2, EDNRA, G3BP2, GNAI2, GNAQ, GNAS, HTR2B, ITGB1BP1, OPRK1, OPRM1, PRKCA, RACK1, YWHAZ* |
|  | Regulation of blood pressure | *ADRB1, ADRB2, AGTR1, AGTRAP, BDKRB2, EDNRA, TBXA2R* |
|  | Negative regulation of cell communication | *ADRB2, ARRB2, BDKRB2, CAV3, CDH2, G3BP2, GNAI2, GPRASP1, GRK5, GRK6, HTR2B, ITGB1BP1, OPRK1, OPRM1, PRKCA, RACK1, RAF1* |
|  | Anatomical structure homeostasis | *ADRB2, CAV3, CDH15, GNAS, WDR36* |
|  | Positive regulation of signal transduction | *ADRB1, ADRB2, ARRB2, CDH15, CDH2, EDNRA, GNAI2, GNAS, HTR2B, ITGB1BP1, OPRK1, OPRM1, PRKCA, RACK1, RAF1, YWHAZ* |
|  | Negative regulation of signal transduction | *ADRB2, ARRB2, BDKRB2, CAV3, CDH2, G3BP2, GPRASP1, GRK5, GRK6, HTR2B, ITGB1BP1, OPRM1, PRKCA, RACK1, RAF1* |
|  | Regulation of cyclase activity | *ADRB1, ADRB2, EDNRA, GNAI2, GNAS, OPRM1, PRKCA, RAF1* |
|  | Regulation of intracellular transport | *EDNRA, G3BP2, GNAQ, ITGB1BP1, PRKCA, RACK1, YWHAZ* |
|  | Regulation of hydrolase activity | *ADRB1, ADRB2, AGTR1, ARRB2, EDNRA, GNA13, GNAQ, GNAS, HTR2B, ITGB1BP1, PRKCA, RACK1, RAF1, TBXA2R* |
|  | Regulation of lyase activity | *ADRB1, ADRB2, EDNRA, GNAI2, GNAS, OPRM1, PRKCA, RAF1* |
|  | Regulation of establishment of protein localization | *G3BP2, GNAI2, GNAQ, GNAS, HTR2B, ITGB1BP1, OPRM1, PRKCA, RACK1, YWHAZ* |
|  | Synaptic signalling | *ADRB2, ARRB2, GNAI2, GNB1, HRH2, KCNMA1, OPRD1, OPRK1, OPRM1, PRKCA, RAF1* |
|  | Regulation of cellular protein localization | *CAV3, CDH2, G3BP2, GNAQ, ITGB1BP1, RACK1, YWHAZ* |
|  | Nucleoside phosphate metabolic process | *ADRB1, ADRB2, EDNRA, GNAI2, GNAS, HTR2B, NME1-NME2, OPRM1, PRKCA, RACK1, RAF1* |
|  | Regulation of G-protein coupled receptor protein signaling pathway | *ADRB2, ARRB2, GNB1, GPRASP1, GRK5, GRK6, HTR2B, PRKCA* |
|  | Positive regulation of cyclase activity | *ADRB1, ADRB2, EDNRA, GNAS, PRKCA, RAF1* |
|  | Regulation of ion transport | *ADRB2, ARRB2, CAV3, EDNRA, GNAI2, GRK6, KCNMA1, OPRD1, OPRK1, OPRM1, PRKCA* |
|  | Positive regulation of hydrolase activity | *ADRB1, ADRB2, AGTR1, EDNRA, GNA13, GNAQ, GNAS, HTR2B, PRKCA, RACK1, TBXA2R* |
|  | Positive regulation of lyase activity | *ADRB1, ADRB2, EDNRA, GNAS, PRKCA, RAF1* |
|  | Cardiovascular system development | *ADRB2, CAV3, CDH2, EDNRA, GNA11, GNA13, GNAQ, HTR2B, ITGB1BP1, PRKCA, RAF1, TBXA2R* |
|  | Regulation of intracellular signal transduction | *ADRB1, ADRB2, ARRB2, BDKRB2, CAV3, CDH2, EDNRA, G3BP2, GNA12, GNAI2, GNAS, HTR2B, ITGB1BP1, OPRK1, OPRM1, PRKCA, RACK1, RAF1* |
|  | Signal release | *AGTR1, GNAI2, GNAS, OPRK1, OPRM1, PRKCA, RAF1* |
|  | Regulation of ion transmembrane transport | *CAV3, EDNRA, GRK6, KCNMA1, OPRK1, OPRM1, PRKCA* |
|  | Negative regulation of G-protein coupled receptor protein signaling pathway | *ADRB2, ARRB2, GPRASP1, GRK5, GRK6, HTR2B* |
|  | Negative regulation of intracellular signal transduction | *BDKRB2, CAV3, G3BP2, ITGB1BP1, OPRM1, PRKCA, RACK1* |
|  | Positive regulation of intracellular signal transduction | *ADRB1, ADRB2, ARRB2, CDH2, EDNRA, GNAI2, GNAS, HTR2B, ITGB1BP1, OPRK1, OPRM1, PRKCA, RACK1, RAF1* |
|  | Blood vessel morphogenesis | *ADRB2, CDH2, EDNRA, GNA13, ITGB1BP1, PRKCA, TBXA2R* |
|  | MAPK cascade | *ADRB2, ARRB2, CAV3, CDH2, EDNRA, GNAI2, HTR2B, ITGB1BP1, OPRK1, OPRM1, PRKCA, RAF1* |
| Response to wounding | Response to wounding | *ADRB2, AGTR1, ARRB2, CAV3, CDH15, EDNRA, GNA11, GNA12, GNA13, GNAI2, GNAQ, GNAS, GNB1, KCNMA1, OPRM1, PRKCA, RAF1, TBXA2R, YWHAZ* |
|  | Regulation of homeostatic process | *AGTR1, CAV3, EDNRA, HTR2B, OPRK1, PRKCA, RACK1* |
|  | Homeostatic process | *ADRB1, ADRB2, AGTR1, BDKRB2, CAV3, CDH15, CDH2, EDNRA, GNA13, GNAI2, GNAS, GNB1, HTR2B, KCNMA1, OPRK1, OPRM1, PRKCA, RACK1, RAF1, TBXA2R, WDR36* |
|  | Muscle contraction | *ADRB2, BDKRB2, CAV3, EDNRA, HTR2B, KCNMA1, PRKCA, TBXA2R* |
|  | Chemical homeostasis | *AGTR1, BDKRB2, CAV3, EDNRA, GNA13, GNAI2, GNAS, GNB1, HTR2B, KCNMA1, OPRM1, PRKCA, RACK1, RAF1, TBXA2R* |
|  | Cellular chemical homeostasis | *AGTR1, BDKRB2, CAV3, EDNRA, GNA13, GNB1, HTR2B, KCNMA1, OPRM1, PRKCA, RACK1, RAF1, TBXA2R* |
| Regulation of hydrolase activity | Muscle contraction | *ADRB2, BDKRB2, CAV3, EDNRA, HTR2B, KCNMA1, PRKCA, TBXA2R* |
|  | Regulation of hydrolase activity | *ADRB1, ADRB2, AGTR1, ARRB2, EDNRA, GNA13, GNAQ, GNAS, HTR2B, ITGB1BP1, PRKCA, RACK1, RAF1, TBXA2R* |
|  | Heart development | *CAV3, EDNRA, GNA11, GNAQ, HTR2B, RAF1* |
|  | Positive regulation of hydrolase activity | *ADRB1, ADRB2, AGTR1, EDNRA, GNA13, GNAQ, GNAS, HTR2B, PRKCA, RACK1, TBXA2R* |
|  | Cardiovascular system development | *ADRB2, CAV3, CDH2, EDNRA, GNA11, GNA13, GNAQ, HTR2B, ITGB1BP1, PRKCA, RAF1, TBXA2R* |
|  | Blood vessel morphogenesis | *ADRB2, CDH2, EDNRA, GNA13, ITGB1BP1, PRKCA, TBXA2R* |
|  | MAPK cascade | *ADRB2, ARRB2, CAV3, CDH2, EDNRA, GNAI2, HTR2B, ITGB1BP1, OPRK1, OPRM1, PRKCA, RAF1* |
| G-protein coupled receptor signaling pathway | G-protein coupled receptor signaling pathway | *ADRB1, ADRB2, AGTR1, AGTRAP, ARRB2, BDKRB2, EDNRA, GNA11, GNA12, GNA13, GNAI2, GNAQ, GNAS, GNB1, GPRASP1, GRK5, GRK6, HRH2, HTR2B, OPRD1, OPRK1, OPRM1, PRKCA, TBXA2R* |

Abbreviation- PTAFR: platelet activating factor receptor.
